# Capivasertib Combination Therapy in Advanced Breast Cancer and Other Solid Tumors: A Systematic Review and Meta-Analysis of Efficacy, Safety, and Biomarker-Defined Outcomes

**DOI:** 10.3390/ph19101614

**Published:** 2026-10-11

**Authors:** Moudi M. Alasmari, Rana M. Aldossari, Marwh G. Aldriwesh, Marwa Qadri, Marey A. Almaghrabi, Rawabi A. Alashari

**Affiliations:** 1 College of Medicine, King Saud Bin Abdulaziz University for Health Sciences (KSAU-HS), Jeddah 21556, Saudi Arabia; 2 King Abdullah International Medical Research Centre (KAIMRC), Jeddah 21556, Saudi Arabia; 3 Ministry of National Guard-Health Affairs (MNGHA), Jeddah 21556, Saudi Arabia; 4 Department of Pharmacology and Toxicology, College of Pharmacy, Prince Sattam Bin Abdelaziz University, Al Kharj 11942, Saudi Arabia; 5 Department of Clinical Laboratory Sciences, College of Applied Medical Sciences, King Saud Bin Abdulaziz University for Health Sciences (KSAU-HS), Riyadh 11481, Saudi Arabia; aldriweshm@ksau-hs.edu.sa; 6 King Abdullah International Medical Research Centre (KAIMRC), Riyadh 11481, Saudi Arabia; 7 Ministry of National Guard-Health Affairs (MNGHA), Riyadh 11426, Saudi Arabia; 8 Department of Pharmacology and Toxicology, College of Pharmacy, Jazan University, Jazan 45142, Saudi Arabia; 9 Health Research Center (HRC), Jazan University, Jazan 45142, Saudi Arabia; 10 Department of Pharmaceutics and Pharmaceutical Industries, College of Pharmacy, Taibah University, Madinah 42353, Saudi Arabia; mmaghrabi@taibahu.edu.sa; 11 Department of Clinical Pharmacology, Faculty of Medicine, King Abdulaziz University, Jeddah 21589, Saudi Arabia

**Keywords:** capivasertib, AKT inhibition, *PI3K*/*AKT*/*PTEN* pathway, advanced breast cancer, solid tumors, meta-analysis

## Abstract

**Background:** Capivasertib is an oral pan-AKT inhibitor evaluated in combination with multiple systemic therapies across solid tumors, but its efficacy and safety vary by disease setting, biomarker status, and treatment backbone. We aimed to systematically evaluate the efficacy, safety, and early-phase clinical activity of capivasertib-containing combination regimens in advanced breast cancer and other solid tumors. **Methods:** MEDLINE, Web of Science, Scopus, Cochrane CENTRAL, trial registries, and major oncology conference sources were searched from inception to 31 May 2026. Randomized comparative studies, single-arm phase II studies, and phase I/Ib combination cohorts were included. Random-effects meta-analyses used restricted maximum likelihood estimation, with randomized and noncomparative evidence synthesized separately. **Results:** Twenty-seven reports representing 23 independent cohorts were included. In hormone receptor-positive/HER2-negative advanced breast cancer, capivasertib plus fulvestrant improved progression-free survival in the overall population (HR 0.59, 95% CI 0.51–0.69) and in *PIK3CA*/*AKT1*/*PTEN*-altered disease (HR 0.49, 95% CI 0.38–0.62), and increased objective response (RR 2.23, 95% CI 1.31–3.80). In triple-negative breast cancer, progression-free survival improved (HR 0.72, 95% CI 0.62–0.84), whereas overall survival remained inconclusive (HR 0.80, 95% CI 0.55–1.17). In *PTEN*-deficient metastatic hormone-sensitive prostate cancer, radiographic progression-free survival improved (HR 0.81, 95% CI 0.66–0.98). Capivasertib increased grade ≥ 3 adverse events (RR 1.66, 95% CI 1.06–2.60) and treatment discontinuation (RR 2.97, 95% CI 1.27–6.96). **Conclusions:** Capivasertib demonstrated disease-, biomarker-, and treatment backbone-specific activity, with the most consistent comparative benefit observed in hormone receptor-positive/HER2-negative advanced breast cancer. Increased toxicity should be considered when selecting patients and treatment combinations.

## 1. Introduction

The phosphatidylinositol 3-kinase/protein kinase B/phosphatase and tensin homolog (*PI3K*/*AKT*/*PTEN*) pathway regulates cellular proliferation, survival, metabolism, and treatment adaptation, and its activation through *PIK3CA* or *AKT1* alterations or *PTEN* loss contributes to disease progression and therapeutic resistance in several solid tumors [1,2]. In estrogen receptor-positive (ER-positive) breast cancer (BC), increased AKT signaling has been associated with endocrine resistance, whereas in prostate cancer, *PTEN* loss promotes reciprocal signaling between the PI3K/AKT and androgen–receptor pathways and has been linked to poorer outcomes with androgen-directed therapy [3,4,5]. Capivasertib is an oral ATP-competitive inhibitor of *AKT1*, *AKT2*, and *AKT3* that suppresses downstream signaling and has demonstrated antitumor activity in preclinical breast and prostate cancer models [6]. Early clinical studies established an intermittent 480-mg twice-daily, 4-days-on/3-days-off schedule, confirmed pharmacodynamic target modulation, and reported preliminary activity in molecularly selected tumors, including *AKT1^E17K^*-mutant ER-positive metastatic breast cancer (MBC) [7,8,9]. Its development subsequently expanded to combination strategies beyond endocrine therapy, including olaparib, with evidence of clinical activity in both BRCA1/2-mutant and wild-type tumors [10].

Randomized clinical evidence has subsequently shown that the efficacy of capivasertib is strongly dependent on disease context and treatment backbone. In hormone receptor-positive (HR-positive)/HER2-negative advanced BC, the addition of capivasertib to paclitaxel did not improve progression-free survival (PFS) in BEECH [11], whereas FAKTION demonstrated improvements in PFS and overall survival (OS) when capivasertib was combined with fulvestrant, with expanded biomarker analyses indicating greater benefit in tumors harboring *PIK3CA*, *AKT1*, or *PTEN* alterations [12]. CAPItello-291 subsequently confirmed a PFS benefit with capivasertib plus fulvestrant in both the overall population and the pathway-altered population after progression during or following aromatase-inhibitor-based therapy [13]. In triple-negative BC, PAKT reported progression-free and overall-survival signals with capivasertib plus paclitaxel [14]; however, the larger phase III CAPItello-290 trial did not meet its prespecified overall-survival endpoints [15].

The prostate cancer program has been similarly heterogeneous. ProCAID did not improve its primary composite PFS endpoint when capivasertib was added to docetaxel and prednisolone, despite an exploratory overall-survival signal [16]. RE-AKT did not demonstrate improved antitumor activity with capivasertib plus enzalutamide after prior abiraterone and docetaxel [17]. By contrast, CAPItello-281 demonstrated improved radiographic PFS with capivasertib plus abiraterone and androgen-deprivation therapy in *PTEN*-deficient metastatic hormone-sensitive prostate cancer [18]. These divergent findings indicate that the effects of *AKT* inhibition cannot be generalized across tumor types or treatment settings and may be influenced by tumor lineage, disease state, biomarker definition, combination partner, previous therapy, pharmacological exposure, and endpoint selection.

The clinical evidence for capivasertib spans multiple malignancies, molecularly selected and unselected populations, diverse treatment backbones, and different phases of drug development. Interpretation across individual studies is complicated by variation in pathway-alteration definitions, outcome measures, analysis populations, survival-data maturity, and toxicity ascertainment. A synthesis that indiscriminately combines these settings could obscure clinically important differences, whereas consideration of individual trials alone does not quantify the consistency or precision of treatment effects within comparable settings. Previous reviews have addressed capivasertib within more limited evidence frameworks. A 2023 meta-analysis included four randomized trials across solid tumors and preceded the availability of subsequent phase III data, while a 2024 network meta-analysis evaluated PI3K and AKT inhibitors at the drug-class level rather than examining capivasertib within clinically distinct disease settings, treatment backbones, and biomarker-defined populations [19,20]. Accordingly, the present systematic review and meta-analysis was designed to provide an updated and clinically stratified synthesis of capivasertib-containing regimens, with separate evaluation of randomized comparative and uncontrolled prospective evidence. Particular emphasis was placed on disease- and backbone-specific efficacy, biomarker-defined outcomes, maturity of survival data, treatment-related toxicity, and early-phase information relevant to dose selection and pharmacokinetic interactions.

## 2. Methods

This systematic review and meta-analysis was conducted in accordance with the Preferred Reporting Items for Systematic Reviews and Meta-Analyses 2020 statement and the methodological guidance of the Cochrane Handbook for Systematic Reviews of Interventions [21,22]. The review protocol was developed before quantitative synthesis and was registered in PROSPERO under registration number CRD420261462047.

### 2.1. Eligibility Criteria

Studies were eligible if they enrolled adults aged ≥18 years with unresectable, locally advanced, recurrent, or metastatic solid tumors and evaluated capivasertib in combination with at least one systemic anticancer treatment. Eligible combination partners included endocrine therapy, chemotherapy, androgen-directed therapy, poly(ADP-ribose) polymerase inhibitors, antibody–drug conjugates, immunotherapy, selective estrogen receptor degraders, and other targeted agents. Eligible designs comprised randomized phase II and III trials, randomized extension cohorts with separately extractable nonoverlapping populations, prospective single-arm phase II studies, and phase I or Ib dose-escalation or dose-expansion studies. Comparative studies were required to include placebo plus the same treatment backbone, the treatment backbone alone, or another protocol-defined standard-of-care regimen. Randomized comparative studies, single-arm phase II studies, and phase I/Ib studies were treated as separate evidence strata. Noncomparative cohorts were included to characterize preliminary antitumor activity, dose selection, safety, and pharmacokinetics; however, they were not pooled with randomized studies to estimate comparative treatment effects. The principal efficacy outcomes were PFS, radiographic PFS, composite PFS, OS, and objective response rate (ORR). Additional efficacy outcomes included clinical benefit rate, disease-control rate, duration of response, time to progression, PFS2, time to subsequent treatment, tumor-marker response, and patient-reported outcomes.

Safety outcomes included any adverse event, grade ≥ 3 adverse events, serious adverse events, adverse-event-related death, adverse events leading to treatment discontinuation, dose interruption, or dose reduction, and individual toxicities. Dose-limiting toxicity, maximum tolerated dose, recommended phase II dose, pharmacokinetic outcomes, and preliminary antitumor activity were additionally extracted from phase I/Ib studies. Studies evaluating capivasertib monotherapy were excluded. Multi-cohort reports containing both monotherapy and combination-treatment cohorts were eligible only when combination-specific data were separately extractable. Preclinical and animal studies, healthy-volunteer studies, case reports, retrospective series without a prospectively defined treatment cohort, reviews, editorials, pharmacometric analyses without independently extractable clinical outcomes, and conference abstracts without a complete peer-reviewed publication were excluded. No language restrictions were applied.

### 2.2. Information Sources and Search Strategy

MEDLINE via PubMed, Web of Science, Scopus, and Cochrane CENTRAL were searched from inception to 31 May 2026. Supplementary searches were conducted in ClinicalTrials.gov, the European Union Clinical Trials Information System and EudraCT, the ISRCTN registry, the AstraZeneca clinical-trial registry, and publicly accessible records from major oncology organisations and conferences, including the American Society of Clinical Oncology, European Society for Medical Oncology, American Association for Cancer Research, and San Antonio Breast Cancer Symposium. The search combined free-text and controlled-vocabulary terms, where available, for “capivasertib,” “AZD5363,” and “AZD-5363.” No outcome restrictions were applied. Reference lists of eligible studies and relevant reviews were examined, and backward and forward citation searching was performed. Trial registrations were cross-checked to identify companion reports, verify study characteristics and prespecified outcomes, and assess participant overlaps. Complete source-specific search strategies are provided in Supplementary Table S1.

### 2.3. Study Selection

Records were imported into EndNote X9 (Clarivate, Philadelphia, PA, USA), and duplicates were removed electronically and manually. All deduplicated records were exported to Rayyan (Rayyan Systems Inc., Cambridge, MA, USA) (Rayyan Systems Inc., Cambridge, MA, USA), where two reviewers (R.M.A. and M.Q.) independently screened titles and abstracts and assessed the full texts of potentially eligible reports. Disagreements were resolved by consensus or consultation with a third reviewer (R.A.A.). Reports describing the same trial were linked using trial identifiers, treatment arms, recruitment periods, enrollment numbers, baseline characteristics, and data-cutoff dates. Companion publications reporting updated survival, safety, subgroup, biomarker, or patient-reported outcomes were not counted as independent studies. Regional extension cohorts were treated as independent only when their participants did not overlap with the corresponding global study population. Report-to-trial linkage and overlap decisions are presented in Supplementary Table S2.

### 2.4. Data Extraction

Data were extracted using a piloted standardized workbook by (R.M.A.) and independently verified by (M.Q.). Discrepancies were resolved by review of the source publication and consensus, with adjudication by (R.A.A.) when required. Extracted study-level variables included trial phase and design, geographical setting, eligibility criteria, disease setting, treatment line, molecular-selection strategy, treatment regimens, capivasertib dose and schedule, sample size, follow-up duration, primary endpoint, and funding source. Participant-level characteristics included age, sex, performance status, prior systemic treatment, metastatic distribution, measurable disease, and biomarker status, where reported.

For efficacy outcomes, hazard ratios (HRs), confidence intervals (CIs), event counts, response numerators and denominators, analysis populations, and data-cutoff dates were extracted. For safety outcomes, event counts, denominators, toxicity grade, treatment attribution, adverse-event definitions, and dose modifications were recorded. Phase I/Ib extraction additionally included dose-limiting toxicities, dose-escalation procedures, maximum tolerated dose, recommended phase II dose, and pharmacokinetic outcomes. Missing values were recorded as not reported and were not interpreted as zero. Reported percentages were recalculated from numerators and denominators when available. When multiple reports provided data for the same outcome and participant cohort, the most complete and mature estimate was selected. Earlier reports were retained when they contributed additional methodological, baseline, safety, or subgroup information.

### 2.5. Risk-of-Bias Assessment

Randomised studies were assessed using the Cochrane Risk of Bias 2 tool (RoB2). Assessments were outcome-specific and considered bias arising from the randomisation process, deviations from intended interventions, missing outcome data, outcome measurement, and selection of the reported result. Overall judgments were categorised as low risk, some concerns, or high risk. The single-arm phase II and phase I/Ib studies were treated as uncontrolled prospective cohorts for evidence interpretation and were appraised using the Joanna Briggs Institute (JBI) Critical Appraisal Checklist for Case Series because they lacked a concurrent comparator and the checklist directly evaluates domains relevant to uncontrolled cohorts, including clear eligibility criteria, consecutive or complete inclusion, outcome ascertainment, follow-up, and reporting [23]. ROBINS-I was prespecified for nonrandomized comparative studies but was not applied because no eligible nonrandomized comparative study was identified. Assessments were performed independently by (R.M.A., M.Q., M.A.A., and R.A.A.), with disagreements resolved by consensus or third-reviewer adjudication.

### 2.6. Effect Measures and Synthesis Eligibility

HRs with 95% CIs were used for time-to-event outcomes. Reported HRs were log-transformed, and standard errors were derived from the confidence limits using the normal quantile corresponding to the originally reported confidence level. Median survival values were not converted into HRs. An HR summarizes the relative event hazard over follow-up and does not directly quantify an absolute difference in survival time. PFS, radiographic PFS, and composite PFS were treated as distinct endpoint definitions and were combined across definitions only in analyses explicitly designated exploratory; OS was analyzed separately. Risk ratios (RRs) with 95% CIs were used for comparative binary outcomes and were calculated from arm-level event counts and denominators where available. For beneficial outcomes such as ORR, an RR > 1 favored capivasertib; for harmful outcomes such as adverse events, an RR < 1 favored capivasertib. ORR reflects the relative probability of tumor response but not response durability. CIs crossing the null value of 1 were interpreted as compatible with no relative difference [22]. Single-arm response and toxicity outcomes were summarized as proportions with 95% CIs.

Studies were pooled only when sufficiently comparable in terms of cancer type, molecular subtype, disease state, treatment line, combination partner, comparator regimen, biomarker-selection strategy, outcome definition, and analysis population. The principal pairwise syntheses evaluated:

Capivasertib plus fulvestrant versus placebo plus fulvestrant in HR-positive/HER2-negative advanced breast cancer;

Capivasertib plus paclitaxel versus placebo plus paclitaxel in first-line advanced or metastatic triple-negative breast cancer.

Other randomized comparisons were presented individually when differences in disease setting, treatment backbone, or endpoint definition precluded clinically meaningful pooling. Analyses combining different cancer types, treatment backbones, or progression-endpoint definitions were designated exploratory.

### 2.7. Certainty of Evidence

The certainty of evidence for the principal randomized comparisons was assessed using the Grading of Recommendations Assessment, Development and Evaluation framework, considering risk of bias, inconsistency, indirectness, imprecision, and publication bias. Certainty was categorized as high, moderate, low, or very low for each comparison and outcome by R.M.A, MQ and R.A.A, with disagreements resolved by consensus.

### 2.8. Statistical Analysis

Analyses were performed in R version 4.6.1 (R Foundation for Statistical Computing, Vienna, Austria) using the meta for package version 5.0-1 and were cross-checked using the meta package version 8.5-1. Random-effects models were used, with between-study variance estimated by restricted maximum likelihood. Inverse-variance weighting was applied to log HRs and log RRs. Hartung–Knapp confidence intervals were used when at least three studies contributed to an analysis. For two-study syntheses, restricted-maximum-likelihood random-effects estimates were reported, with fixed-effect estimates examined in sensitivity analyses. Outcomes reported by a single eligible study were presented without pooling. Single-arm proportions were synthesized using random-effects logistic-normal generalized linear mixed models. Zero-event and 100%-event cohorts were retained when model estimation permitted. Sparse comparative outcomes were analyzed using generalized linear mixed models or Mantel–Haenszel methods, as appropriate. Statistical heterogeneity was assessed using Cochran’s Q, I^2^, and τ^2^. For clinically interpretable random-effects syntheses with at least five contributing studies and meaningful between-study variation, 95% prediction intervals were additionally reported to describe the dispersion of effects expected across comparable settings; prediction intervals were not emphasized for two-study syntheses or very small syntheses in which between-study variance was poorly estimated [22]. Sensitivity analyses compared random-effects and fixed-effect models, conventional and Hartung–Knapp confidence intervals, inverse-variance and Mantel–Haenszel methods, and RR- and odds-ratio-based analyses. Additional analyses evaluated the exclusion of immature survival estimates, regional extension cohorts, and studies at high risk of bias, where applicable. Low-risk-only sensitivity pooling was considered when at least two studies would remain after exclusion of studies with some concerns. Leave-one-out analyses were performed when at least three studies contributed to an outcome. Exploratory analyses examined results according to cancer type, study phase, treatment line, treatment backbone, and biomarker status. Meta-regression was not performed because fewer than 10 clinically comparable studies were available for any outcome. Formal funnel-plot asymmetry tests were not performed because fewer than 10 studies contributed to each principal synthesis. Potential publication and selective-reporting biases were assessed qualitatively by comparing trial registrations, protocols, and published outcomes. All tests were two-sided, and *p* < 0.05 was considered statistically significant.

## 3. Results

### 3.1. Study Selection

**Figure 1:**
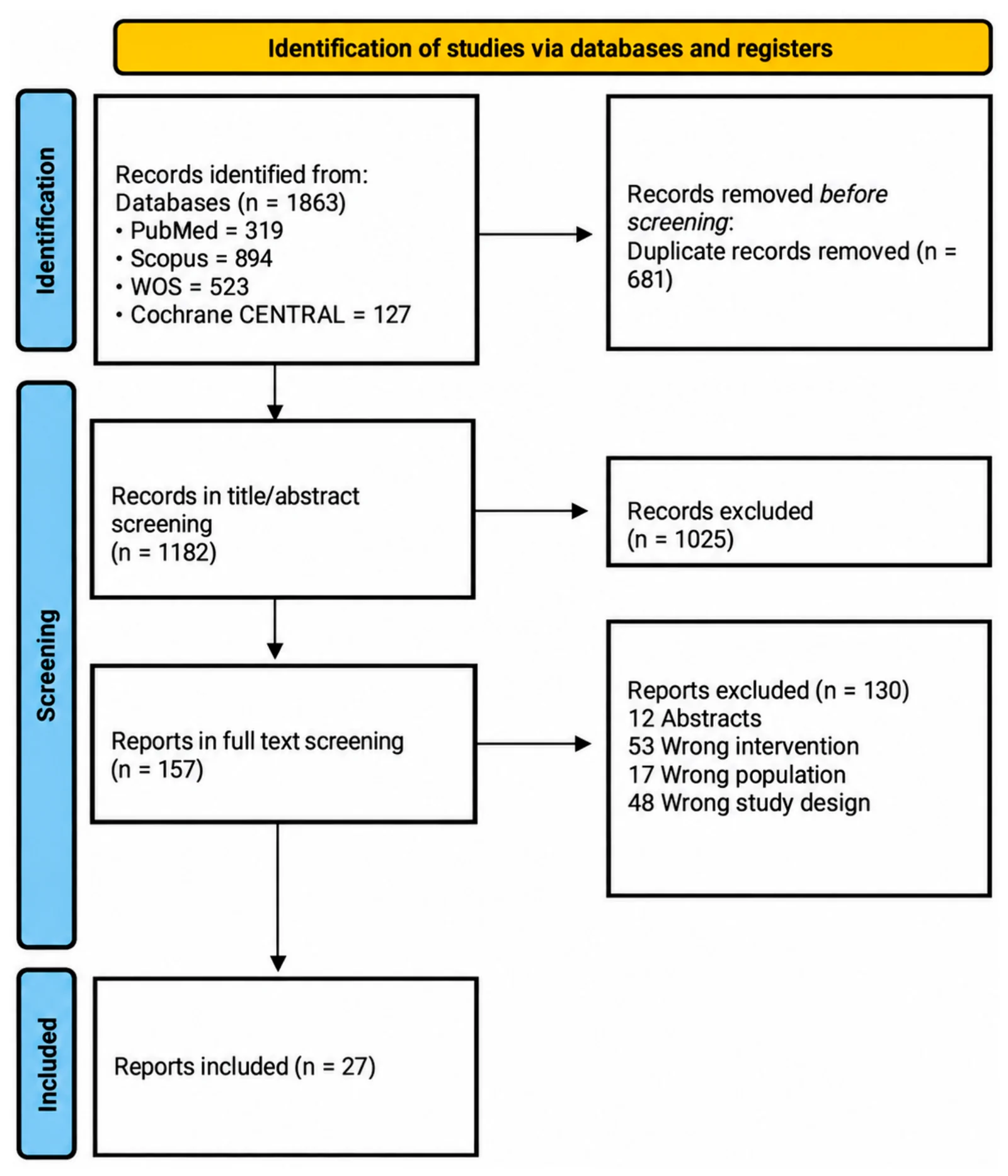
The PRISMA flow diagram.

The database and supplementary searches identified 1863 records. After duplicate removal, 1182 records underwent title and abstract screening, and 157 reports were assessed in full text. One-hundred thirty full-text reports were excluded, leaving 27 eligible reports representing 23 independent analytic cohorts [8,9,10,11,12,13,14,15,16,17,18,24,25,26,27,28,29,30,31,32,33,34,35,36,37,38,39]. These comprised nine randomized comparative cohorts, two single-arm phase II cohorts, and 12 phase I or phase Ib cohorts. Companion publications reporting updated survival, safety, subgroup, regulatory, or patient-reported outcomes were linked to their parent trials and were not counted as independent studies. The study-selection process and reasons for full-text exclusion are presented in Figure 1.

### 3.2. Study, Treatment, and Patient Characteristics

#### 3.2.1. Randomized Comparative Studies

Nine randomized comparative studies were included: six in advanced breast cancer and three in prostate cancer, with sample sizes ranging from 100 participants in RE-AKT to 1012 in CAPItello-281. The breast cancer studies evaluated HR-positive/HER2-negative disease treated with fulvestrant or paclitaxel and first-line metastatic triple-negative breast cancer treated with paclitaxel. The prostate cancer studies evaluated *PTEN*-deficient metastatic hormone-sensitive disease treated with abiraterone and androgen-deprivation therapy and metastatic castration-resistant prostate cancer (mCRPC) disease treated with enzalutamide or docetaxel and prednisolone. Median age ranged from 51.9 to 62 years in breast cancer and from 67 to 72.3 years in prostate cancer. Performance status 0 was reported in 49.2–75.0% and 58.7–64.9% of participants, respectively.

Capivasertib was generally administered at 400 mg twice daily on a 4-days-on/3-days-off schedule, although ProCAID used 320 mg twice daily and dosing was adapted to the chemotherapy schedule in some paclitaxel-based trials.

The randomized populations differed substantially in molecular selection, disease burden, and prior therapy. In CAPItello-291, 40.8% had *PIK3CA*/*AKT1*/*PTEN*-altered tumors, approximately 69% had received a CDK4/6 inhibitor, and 18% had received chemotherapy for advanced disease. BEECH Part B enrolled an ER-positive/HER2-negative population, with *PIK3CA* mutations in 48.1% and 44.6% of the experimental and control groups, prior taxane exposure in 35.2% and 39.3%, and prior hormonal therapy in 79.6% and 69.6%, respectively. Pathway alterations were present in 30.7% of CAPItello-290, 20.0% of PAKT, and 54.3% of FAKTION participants using its expanded biomarker definition. CAPItello-281 enrolled a high-burden *PTEN*-deficient population, with bone metastases in 91.1–92.5% and high-risk disease in 61.3–65.9%. RE-AKT and ProCAID enrolled previously treated mCRPC populations, with bone metastases in 81.3–94.0% and previous androgen–receptor-directed therapy in approximately two-thirds of ProCAID participants. Detailed characteristics are presented in Table 1 and Supplementary Tables S3 and S4.

#### 3.2.2. Single-Arm Phase II and Phase I/Ib Studies

The noncomparative evidence comprised two single-arm phase II cohorts and 12 phase I or Ib cohorts, including 416 treated participants. Cohort size ranged from 10 in ProCAID phase I to 64 in ComPAKT. Seven cohorts evaluated breast cancer, three evaluated prostate cancer, and four were mixed or basket solid-tumour studies. Combination partners included fulvestrant, camizestrant, paclitaxel, docetaxel, abiraterone, olaparib, durvalumab plus paclitaxel, and trastuzumab deruxtecan. Several cohorts were enriched for *AKT1*, *PTEN*, *BRCA1/2*, DNA-damage-response, or broader PI3K/AKT-pathway alterations. Reported median or mean age ranged from 51 to 70.4 years, while performance status 0 ranged from 0% to 75%. Visceral disease was present in 61.3–100% of several breast cancer cohorts, and bone metastases occurred in 81.3% of RE-AKT phase I and 90.0% of ProCAID phase I participants.

Most molecularly selected cohorts enrolled heavily pretreated patients. In plasmaMATCH cohort C, 83.3% had received chemotherapy, all had received endocrine therapy, and 33.3% had received a CDK4/6 inhibitor. Participants in the *PTEN*-mutant fulvestrant cohorts had received a median of seven previous regimens, whereas 89.7% of SERENA-1 participants had received a CDK4/6 inhibitor and 55.2% had received fulvestrant. All RE-AKT phase I participants had received docetaxel, and 87.5% had received abiraterone. In the capivasertib–abiraterone cohort, 81.5% had received docetaxel and 66.7% enzalutamide. ComPAKT participants had received a median of four previous systemic lines, with BRCA1/2-mutant disease in 39.1%, while the olaparib–capivasertib expansion cohort had received a median of three lines and included 18.4% with germline BRCA1/2 mutations. These studies primarily assessed dose-limiting toxicity, pharmacokinetics, dose selection, tolerability, and preliminary activity; their principal characteristics are summarized in Table 2, with detailed baseline data in Supplementary Tables S3 and S4.

### 3.3. Risk of Bias and Methodological Quality

#### 3.3.1. Randomised Studies

Eighteen outcome-specific RoB 2 assessments were completed across the randomized cohorts. Nine assessments were judged to be at low overall risk of bias, and nine had some concerns; none were classified as being at high risk of bias. All assessments were judged to be at low risk for deviations from intended interventions. Concerns were most frequently identified in the domain addressing the selection of the reported result, which affected nine assessments. Less frequent concerns involved the randomization process, missing outcome data, and outcome measurement.

**Figure 2:**
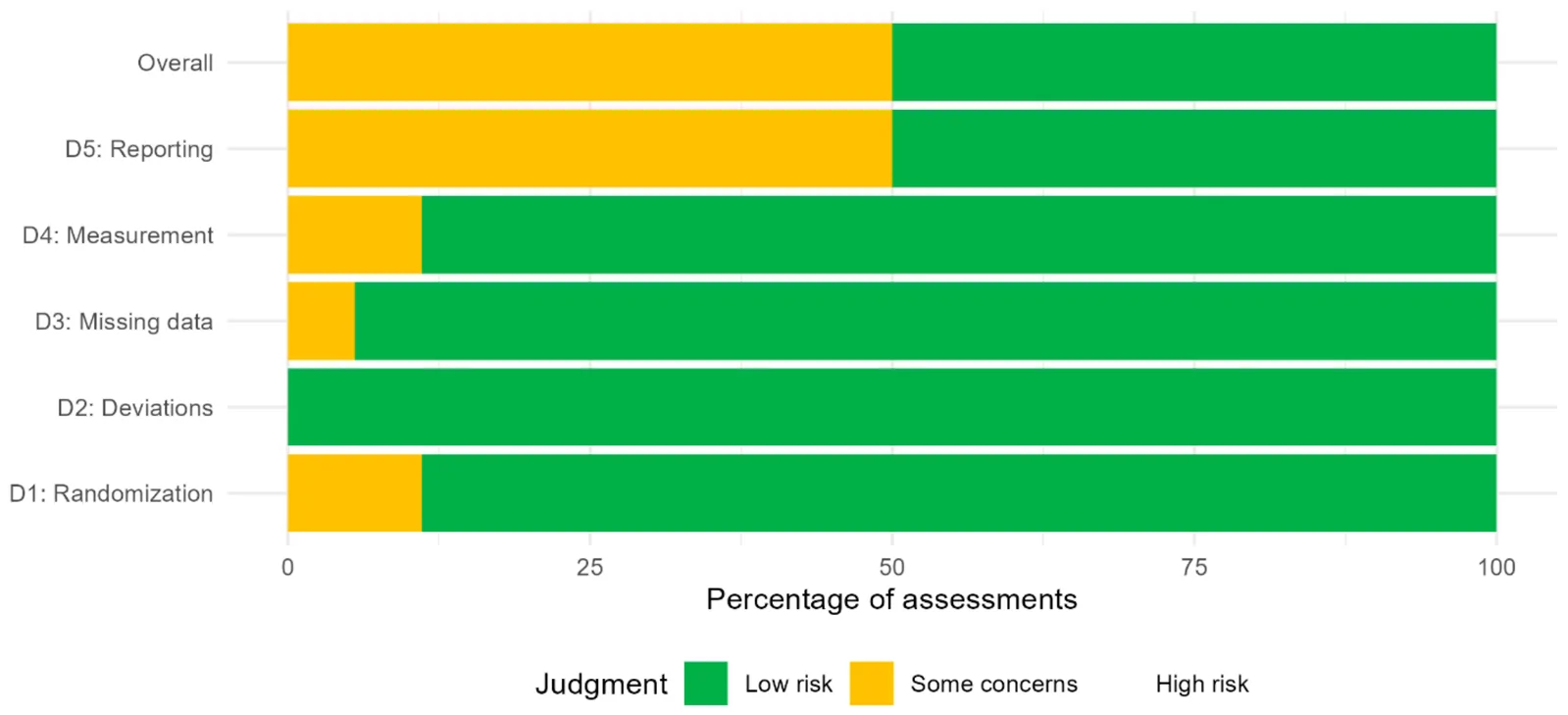
RoB 2 domain-level summary. RoB 2, Cochrane Risk of Bias 2 tool.

CAPItello-291, CAPItello-281, and CAPItello-290 were generally judged to be at low risk of bias for their principal efficacy and safety outcomes. Some concerns were assigned to the Chinese CAPItello-291 extension because of overlapping reporting and selective availability of extension-specific outcomes. The smaller phase II studies had concerns related primarily to incomplete reporting of randomization procedures, outcome measurement, missingness, or selective reporting. The study-level traffic-light plot and domain-level summary are presented in Figure 2 and Supplementary Table S5.

#### 3.3.2. Uncontrolled Studies

The uncontrolled prospective single-arm phase II and phase I/Ib cohorts were appraised using the JBI Critical Appraisal Checklist for Case Series. The principal limitations were the absence of concurrent controls, small cohort sizes, variable completeness of follow-up, selective molecular enrichment, and inconsistent reporting of consecutive or complete participant inclusion. Detailed item-level judgments are provided in Supplementary Table S6.

### 3.4. Comparative Efficacy of Capivasertib-Containing Regimens

Randomized efficacy data were synthesized within clinically coherent disease- and backbone-specific strata. Comparisons that differed materially in tumour subtype, disease state, treatment backbone, biomarker selection, or endpoint definition were reported individually, and broader pooled estimates were designated exploratory.

#### 3.4.1. HR-Positive/HER2-Negative Advanced Breast Cancer

**Figure 3:**
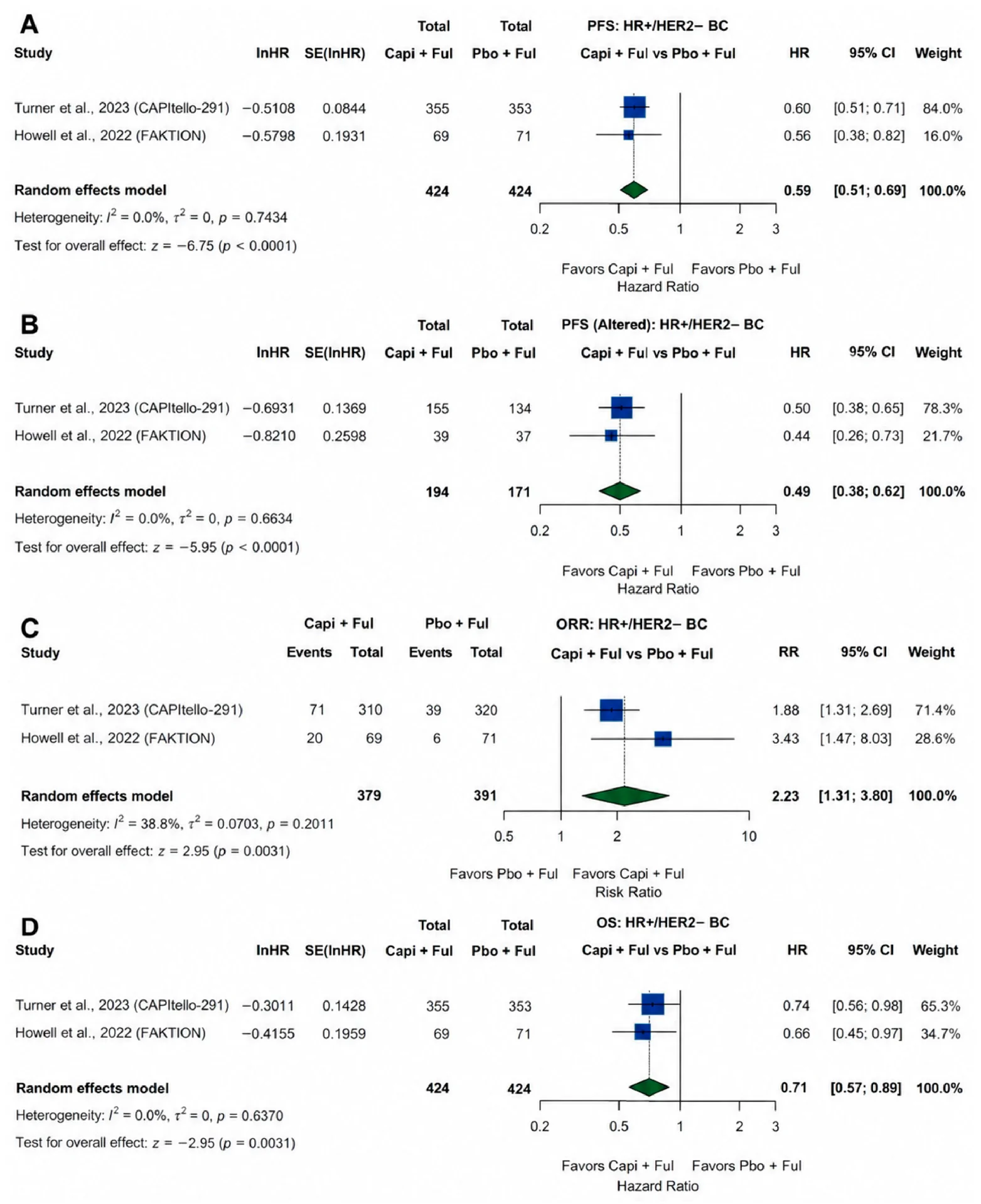
Capivasertib plus fulvestrant in hormone receptor-positive/HER2-negative advanced breast cancer. (**A**) Progression-free survival (PFS) in the overall population. (**B**) PFS in the *PIK3CA*/*AKT1*/*PTEN*-altered population. (**C**) Objective response rate (ORR). (**D**) Overall survival (OS) in the overall population. HR—hazard ratio; RR—risk ratio. HR < 1 favors capivasertib for time-to-event outcomes; RR > 1 favors capivasertib for objective response (ORR). Squares represent study-specific estimates, with square size proportional to study weight; horizontal lines indicate 95% CIs, and diamonds represent pooled random-effects estimates. The solid vertical line denotes the null value (HR or RR = 1), and the dashed vertical line denotes the pooled random-effects estimate. Data are from FAKTION [12] and CAPItello-291 [13].

Two randomised trials, CAPItello-291 and FAKTION, contributed to the fulvestrant-backbone synthesis. Among 848 participants in the overall intention-to-treat population, capivasertib plus fulvestrant was associated with longer PFS than placebo plus fulvestrant (HR 0.59, 95% CI 0.51–0.69; *p* < 0.001), with no detected heterogeneity (I^2^ = 0%; tau^2^ = 0). In 365 participants with *PIK3CA*/*AKT1*/*PTEN*-altered tumors, the pooled PFS estimate was HR 0.49 (95% CI 0.38–0.62; *p* < 0.001; I^2^ = 0%). Objective response was also more frequent with capivasertib among 770 response-evaluable participants (RR 2.23, 95% CI 1.31–3.80; *p* = 0.003; I^2^ = 38.8%). Mature overall-survival data were available from FAKTION, in which capivasertib plus fulvestrant was associated with longer OS (HR 0.66, 95% CI 0.45–0.97; *p* = 0.034). BEECH Part B evaluated a different chemotherapy backbone and was therefore not pooled with the fulvestrant studies. In 110 participants with ER-positive/HER2-negative advanced breast cancer, capivasertib plus paclitaxel did not significantly improve PFS in the overall population (HR 0.80; *p* = 0.308) or in the *PIK3CA*-mutant subgroup (HR 1.11, 80% CI 0.73–1.68; *p* = 0.760). Inclusion of the immature CAPItello-291 OS estimate with mature FAKTION OS yielded HR 0.71 (95% CI 0.57–0.89; *p* = 0.003; I^2^ = 0%), as shown in Figure 3.

#### 3.4.2. Triple-Negative Breast Cancer

**Figure 4:**
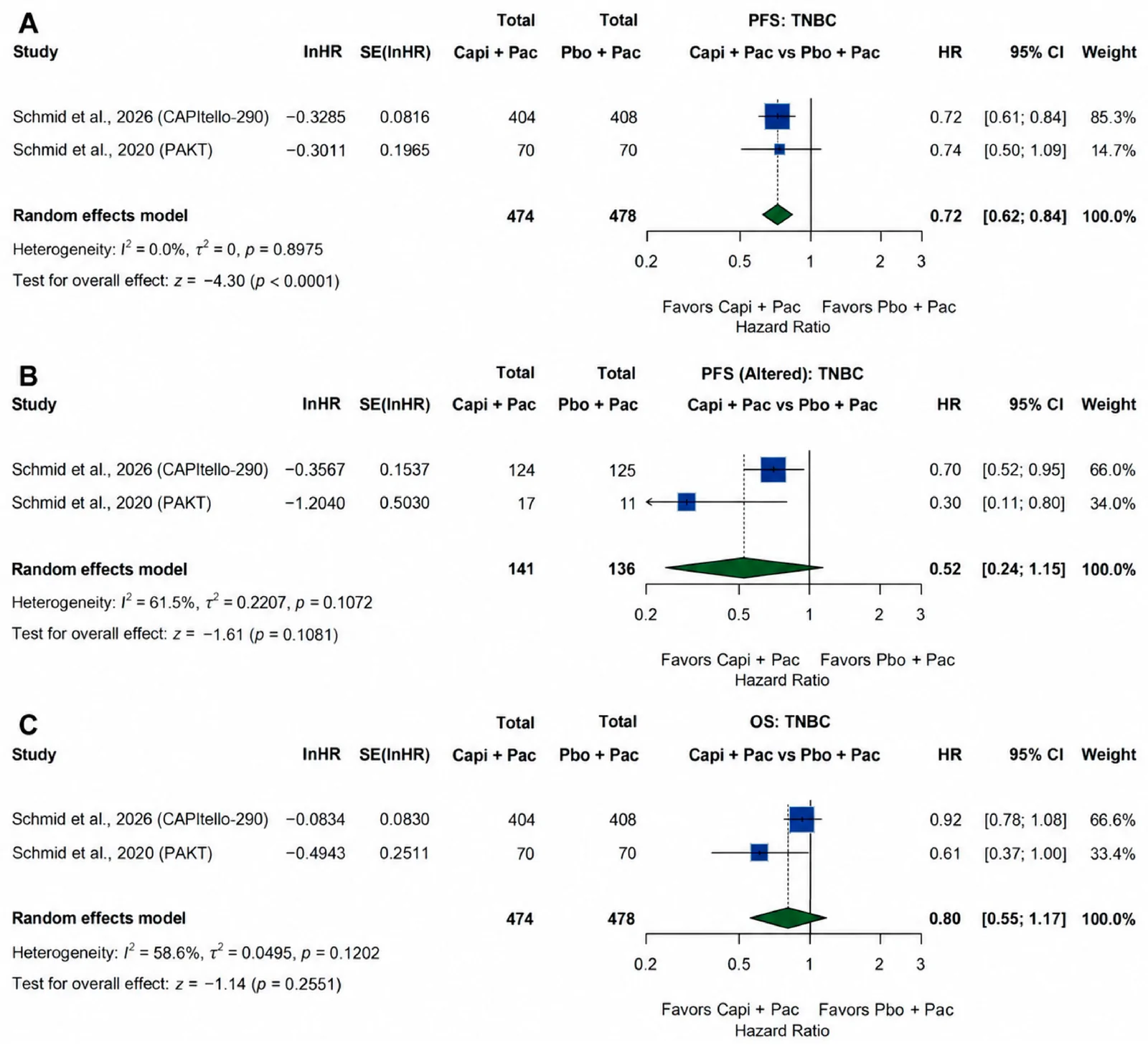
Capivasertib plus paclitaxel in advanced or metastatic triple-negative breast cancer (TNBC). (**A**) Progression-free survival (PFS) in the overall population. (**B**) PFS in the *PIK3CA*/*AKT1*/*PTEN*-altered population. (**C**) Overall survival (OS) in the overall population. HR—hazard ratio; HR < 1 favors capivasertib. Squares represent study-specific estimates, with square size proportional to study weight; horizontal lines indicate 95% CIs, and diamonds represent pooled random-effects estimates. The solid vertical line denotes the null value (HR = 1), and the dashed vertical line denotes the pooled random-effects estimate; an arrow indicates a confidence interval extending beyond the plotted x-axis range. Data are from PAKT [14] and CAPItello-290 [15].

CAPItello-290 and PAKT contributed 952 participants to the first-line advanced or metastatic triple-negative breast cancer synthesis. Capivasertib plus paclitaxel was associated with longer PFS than placebo plus paclitaxel (HR 0.72, 95% CI 0.62–0.84; *p* < 0.001), with no detected heterogeneity (I^2^ = 0%; τ^2^ = 0). Among 277 participants with *PIK3CA*/*AKT1*/*PTEN*-altered tumors, pooled estimates were below 1 but did not reach statistical significance for PFS (HR 0.52, 95% CI 0.24–1.15; *p* = 0.108; I^2^ = 61.5%) or OS (HR 0.72, 95% CI 0.27–1.92; *p* = 0.512; I^2^ = 67.9%). Both confidence intervals crossed the null value and were accompanied by moderate-to-substantial heterogeneity; therefore, these data do not establish a biomarker-specific benefit in TNBC. The pooled OS estimate favored capivasertib but was imprecise and heterogeneous (HR 0.80, 95% CI 0.55–1.17; *p* = 0.255; I^2^ = 58.6%; τ^2^ = 0.0495) (Figure 4).

#### 3.4.3. Prostate Cancer

CAPItello-281 evaluated capivasertib plus abiraterone, corticosteroid, and androgen-deprivation therapy in 1012 participants with *PTEN*-deficient metastatic hormone-sensitive prostate cancer. Capivasertib was associated with longer radiographic PFS (HR 0.81, 95% CI 0.66–0.98; *p* = 0.034), whereas the interim OS estimate remained inconclusive (HR 0.90, 95% CI 0.71–1.15; *p* = 0.401). The two mCRPC trials did not demonstrate statistically significant improvements in their primary efficacy outcomes. In RE-AKT (n = 100), capivasertib plus enzalutamide yielded a composite-response RR of 1.02 (95% CI 0.44–2.35; *p* = 0.580), an rPFS HR of 0.78 (95% CI 0.47–1.30; *p* = 0.330), and an OS HR of 0.76 (95% CI 0.51–1.15; *p* = 0.190). In ProCAID (n = 150), capivasertib plus docetaxel and prednisolone yielded a composite-PFS HR of 0.92 (95% CI 0.73–1.16; *p* = 0.320) and an updated OS HR of 0.70 (95% CI 0.47–1.05; *p* = 0.090).

**Figure 5:**
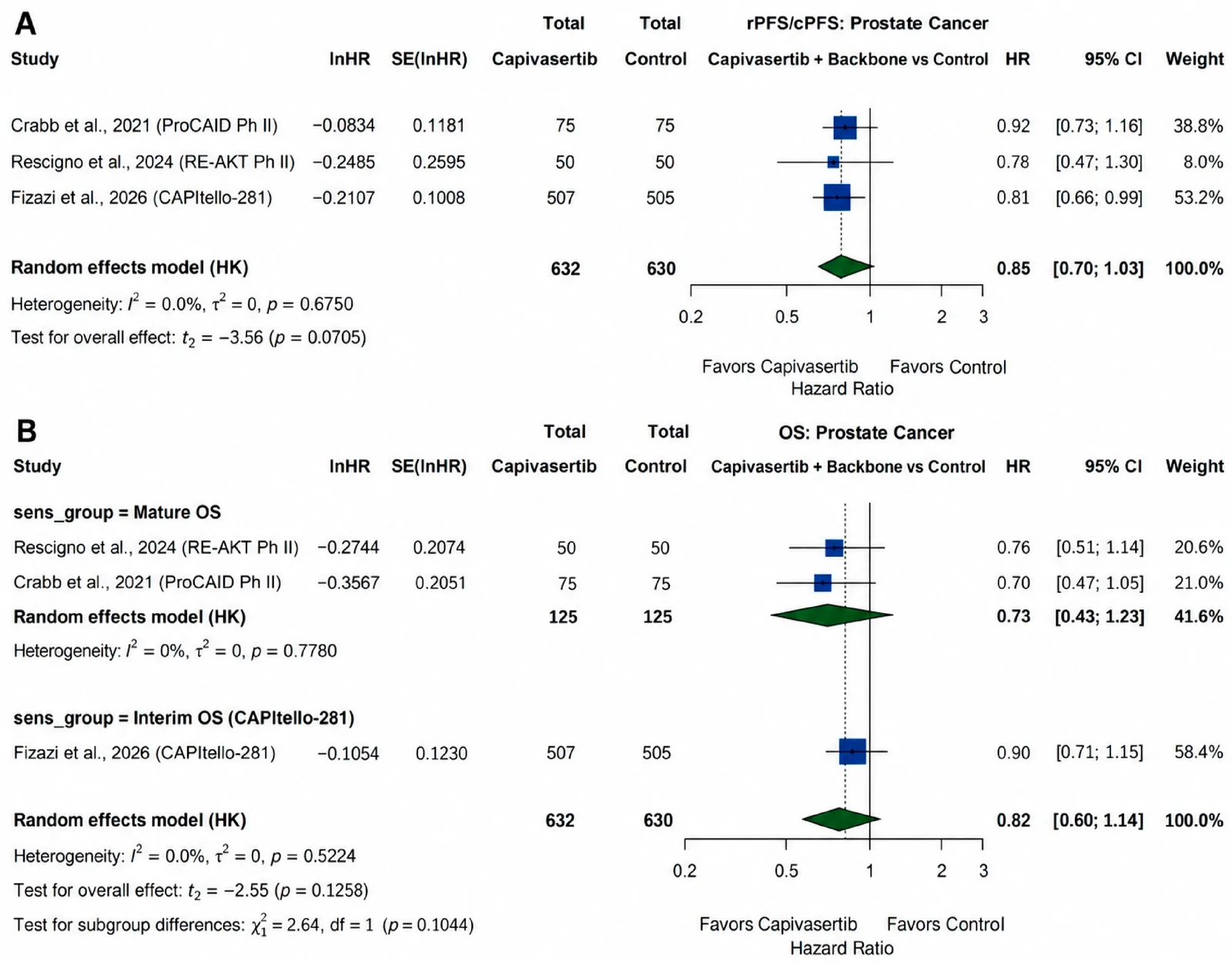
Randomized efficacy in prostate cancer. (**A**) Trial-specific progression outcomes and the exploratory pooled progression estimate across CAPItello-281, RE-AKT, and ProCAID. (**B**) Overall-survival (OS) estimates, including the mature metastatic castration-resistant prostate cancer (mCRPC) synthesis and a sensitivity estimate incorporating interim CAPItello-281 OS. rPFS—radiographic progression-free survival; cPFS—composite progression-free survival; HR—hazard ratio. Pooled estimates are exploratory because disease state, treatment backbone, biomarker selection, and endpoint definitions differed. HR < 1 favors capivasertib. Squares represent study-specific estimates, with square size proportional to study weight; horizontal lines indicate 95% CIs, and diamonds represent pooled random-effects estimates. The solid vertical line denotes the null value (HR = 1), and the dashed vertical line denotes the pooled random-effects estimate. Data are from Pro-CAID [16], RE-AKT [17], and CAPItello-281 [18].

An exploratory synthesis of progression endpoints across CAPItello-281, RE-AKT, and ProCAID produced an HR of 0.85 (95% CI 0.70–1.03; I^2^ = 0%). The mature mCRPC OS estimate from RE-AKT and ProCAID was HR 0.73 (95% CI 0.43–1.23; I^2^ = 0%); inclusion of interim CAPItello-281 OS yielded HR 0.82 (95% CI 0.60–1.14; I^2^ = 0%), as shown in Figure 5.

### 3.5. Comparative Safety in Randomized Studies

#### 3.5.1. Overall Safety Outcomes

**Figure 6:**
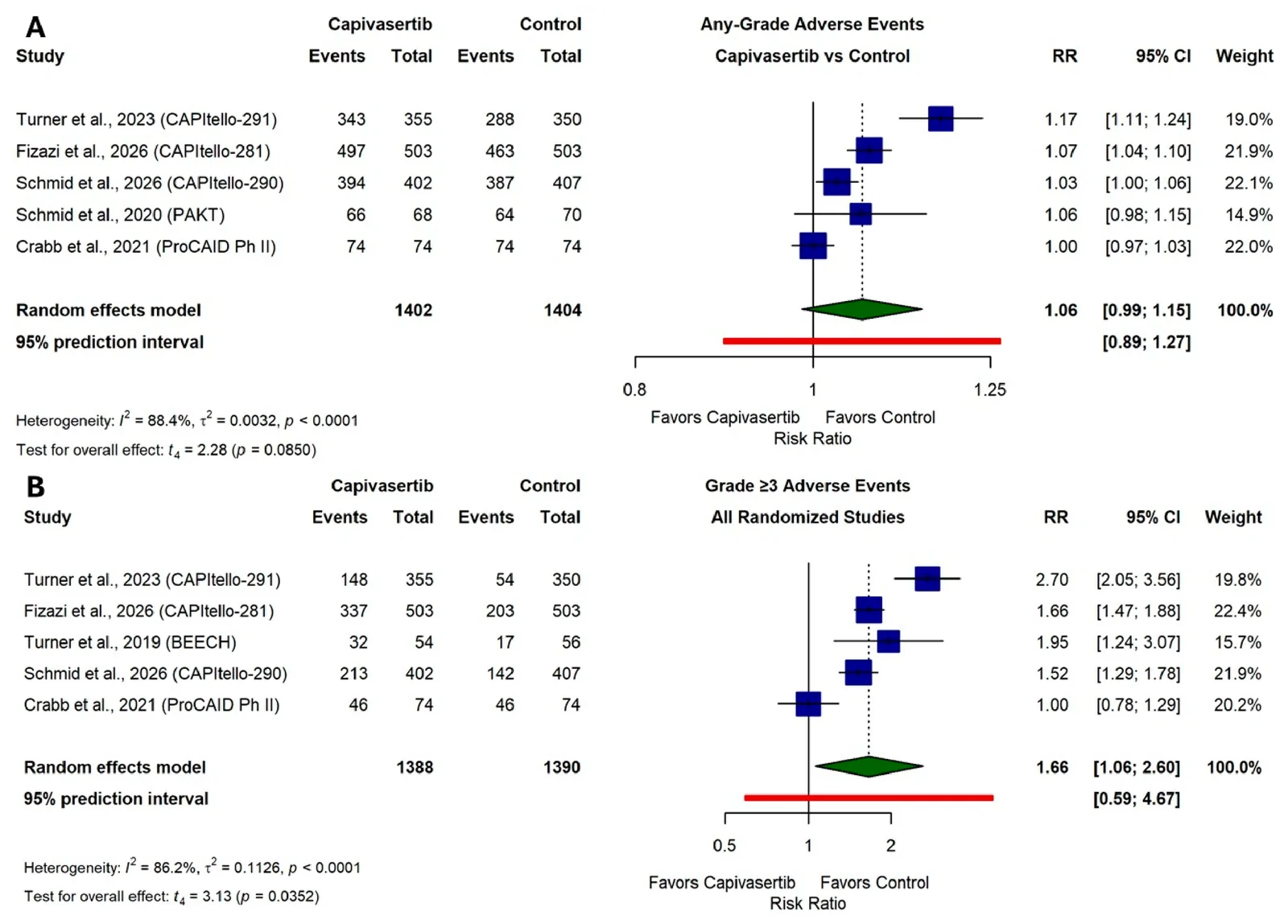

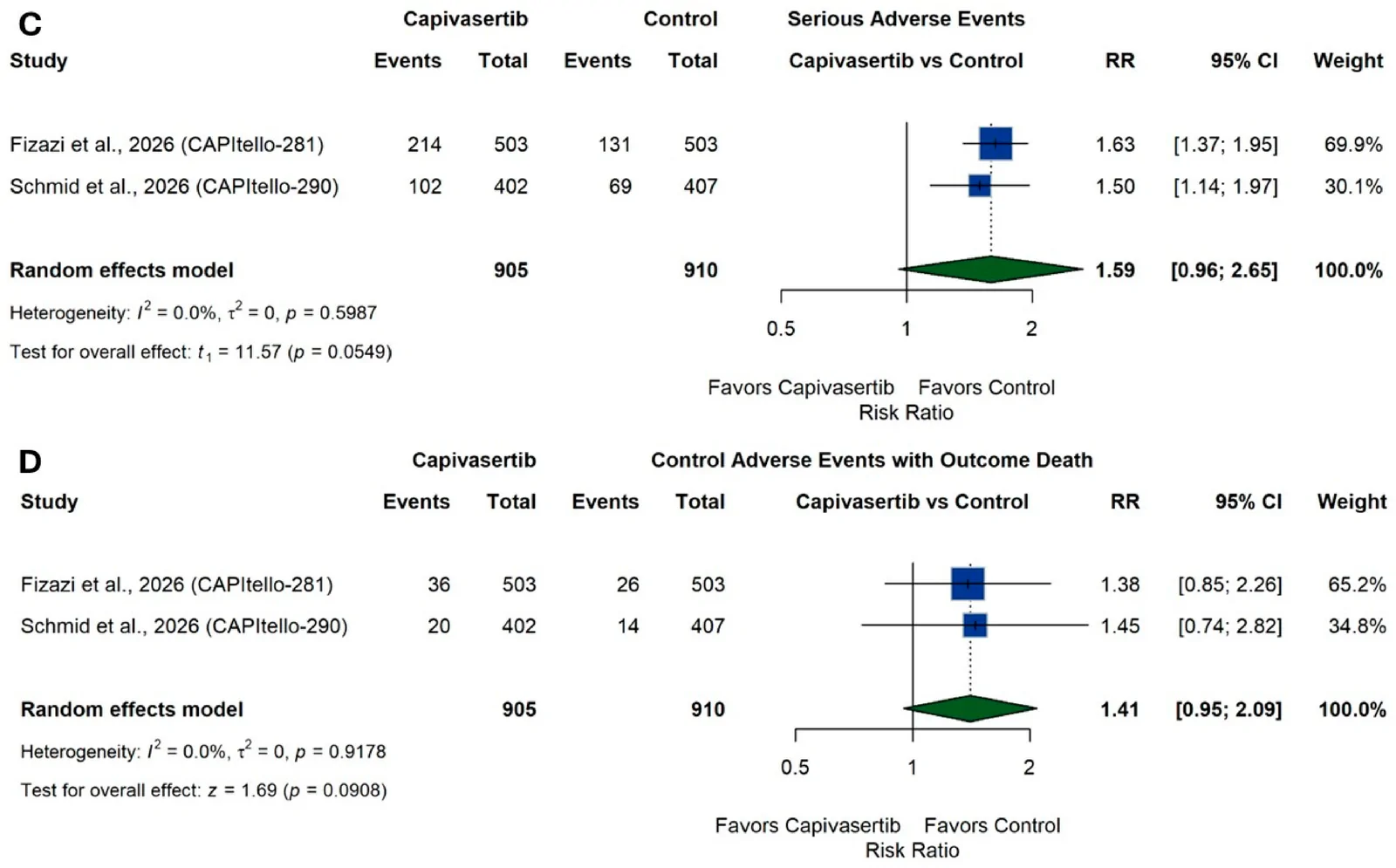
Comparative safety of capivasertib-containing regimens versus control in randomized studies. Forest plots show risk ratios (RRs) for (**A**) any-grade adverse events, (**B**) grade ≥ 3 adverse events, (**C**) serious adverse events, and (**D**) adverse events with an outcome of death. Pooled estimates were calculated using random-effects models and are presented with 95% confidence intervals (CIs). Squares represent study-specific effect estimates, with square size proportional to random-effects weight, and horizontal lines indicate 95% CIs. Red horizontal bars in panels A and B represent 95% prediction intervals. Diamonds represent pooled estimates. An RR > 1 indicates a higher risk with capivasertib, whereas an RR < 1 indicates a lower risk with capivasertib. Heterogeneity was assessed using the I^2^ and between-study variance (τ^2^). The solid vertical line denotes the null value (RR = 1), and the dashed vertical line denotes the pooled random-effects estimate. Data are from BEECH [11], CAPItello-291 [13], PAKT [14], CAPItello-290 [15], ProCAID [16], and CAPItello-281 [18].

Five randomized studies contributed to the any-grade and grade ≥ 3 adverse-events analyses, comprising 2806 participants for any-grade adverse events and 2778 participants for grade ≥ 3 adverse events. The risk of experiencing any adverse event was not statistically different between groups (RR 1.06, 95% CI 0.99–1.15; *p* = 0.085; I^2^ = 88.4%; 95% prediction interval 0.89–1.27). Capivasertib-containing regimens were associated with a higher average risk of grade ≥ 3 adverse events (RR 1.66, 95% CI 1.06–2.60; *p* = 0.035; I^2^ = 86.2%; 95% prediction interval 0.59–4.67) and adverse events leading to treatment discontinuation (four studies; 2668 participants; RR 2.97, 95% CI 1.27–6.96; *p* = 0.027; I^2^ = 66.7%). The substantial heterogeneity indicates that these pooled safety RRs represent average effects and should not be interpreted as uniform increases applicable to every disease setting or treatment backbone. Estimates for serious adverse events (two studies; 1815 participants; RR 1.59, 95% CI 0.96–2.65; *p* = 0.055; I^2^ = 0%) and adverse-event-related death (RR 1.41, 95% CI 0.95–2.09; *p* = 0.091; I^2^ = 0%) were imprecise and did not reach statistical significance (Figure 6).

#### 3.5.2. Specific Adverse Events

The excess toxicity was concentrated in established on-target adverse events. Any-grade diarrhea (six studies; RR 2.78, 95% CI 1.51–5.11; *p* = 0.008; I^2^ = 94.8%), rash (six studies; RR 3.61, 95% CI 2.41–5.40; *p* < 0.001; I^2^ = 68.8%), and hyperglycaemia (five studies; RR 2.87, 95% CI 2.10–3.91; *p* < 0.001; I^2^ = 22.2%) occurred more frequently with capivasertib. The relative increases were larger for grade ≥ 3 or source-defined severe diarrhea (eight studies; RR 8.21, 95% CI 3.59–18.79; *p* < 0.001; I^2^ = 40.8%), rash (six studies; RR 23.47, 95% CI 9.10–60.56; *p* < 0.001; I^2^ = 0%), and hyperglycemia (six studies; RR 12.30, 95% CI 7.06–21.42; *p* < 0.001; I^2^ = 0%). Pooled estimates for any-grade fatigue (RR 1.22, 95% CI 0.87–1.72), neutropenia (RR 1.20, 95% CI 0.68–2.10), and nausea (RR 1.59, 95% CI 0.66–3.85) were not statistically significant (Table 3).

### 3.6. Noncomparative Phase II and Phase I/Ib Evidence

#### 3.6.1. Antitumor Activity

Nine eligible noncomparative cohorts comprising 252 response-evaluable participants contributed to the pooled ORR analysis. The pooled ORR was 25% (95% CI 12–44%; I^2^ = 72.2%; 95% prediction interval 3–78%), indicating substantial variability in response across comparable single-arm settings; evidence of variation by combination-partner class was also observed (subgroup *p* < 0.0001), as shown in Figure S1. Across three biomarker-selected ER-positive breast cancer cohorts treated with capivasertib plus fulvestrant (n = 92), the pooled ORR was 24% (95% CI 10–47%; I^2^ = 0%). Seven cohorts comprising 225 participants contributed to the clinical-benefit-rate synthesis, which yielded a pooled CBR of 43% (95% CI 35–51%; I^2^ = 0%) without evidence of variation by partner class (subgroup *p* = 0.575), as shown in Figure S2.

#### 3.6.2. Early-Phase Safety, Dose Selection, and Pharmacokinetics

Seven single-arm cohorts comprising 212 participants contributed to the grade ≥ 3 adverse-event synthesis, with a pooled proportion of 63% (95% CI 50–73%; I^2^ = 50.9%; 95% prediction interval 38–82%), as shown in Figure S3. Across four dose-finding cohorts comprising 97 participants, the pooled dose-limiting-toxicity proportion was 9% (95% CI 3–24%; I^2^ = 0%), as shown in Figure S4. Dose-finding studies generally supported intermittent capivasertib administration: 400 mg twice daily on a 4-days-on/3-days-off schedule was selected for several endocrine, paclitaxel, and targeted-therapy combinations, whereas 320 mg twice daily on the same schedule was selected with docetaxel and prednisolone in ProCAID phase I. In RE-AKT phase I, 400 mg twice daily was selected with enzalutamide, although enzalutamide reduced capivasertib exposure. These early-phase findings informed regimen selection but remained combination-specific.

### 3.7. Sensitivity Analyses

The principal PFS estimates were unchanged under fixed-effect modelling for both the fulvestrant stratum (HR 0.59, 95% CI 0.51–0.69) and the triple-negative breast cancer stratum (HR 0.72, 95% CI 0.62–0.84). For TNBC OS, the fixed-effect estimate was HR 0.88 (95% CI 0.76–1.03), compared with HR 0.80 (95% CI 0.55–1.17) under the random-effects model, reflecting the effect of between-study heterogeneity on precision. The fulvestrant-stratum ORR result remained directionally and statistically consistent when expressed as an odds ratio (OR 2.67, 95% CI 1.39–5.14) or estimated using Mantel–Haenszel methods (RR 2.09, 95% CI 1.50–2.90). No randomized outcome was judged at high overall risk of bias. For the principal two-study efficacy syntheses, exclusion of the phase II study with some concerns would leave a single phase III trial and therefore would not yield a pooled low-risk-only sensitivity estimate; the remaining phase III PFS estimates were directionally concordant with the pooled results. Full sensitivity analyses are provided in Supplementary Table S7.

### 3.8. Subgroup and Effect-Modification Analyses

Across seven randomized studies comprising 3062 participants, the exploratory pooled analysis of progression outcomes favored capivasertib-containing regimens (HR 0.73, 95% CI 0.62–0.85; I^2^ = 50.6%; *p* = 0.003). When stratified by treatment line, the pooled estimate was HR 0.75 (95% CI 0.64–0.89; I^2^ = 0%) across three first-line studies involving 1964 participants and HR 0.70 (95% CI 0.48–1.02; I^2^ = 70.1%) across four later-line studies involving 1098 participants. The test for subgroup differences did not indicate variation in treatment effect according to treatment line (*p*-interaction = 0.553; Supplementary Figure S5). When stratified by study phase, the pooled estimate was HR 0.70 (95% CI 0.48–1.02; I^2^ = 63.8%) across three phase III studies involving 2532 participants and HR 0.76 (95% CI 0.54–1.08; I^2^ = 39.4%) across four phase II studies involving 530 participants. No statistically significant difference was identified between phase II and phase III studies (*p*-interaction = 0.566; Supplementary Figure S6).

### 3.9. Exploratory Broader Syntheses

Broader analyses that combined different disease settings or treatment backbones were directionally consistent with the principal comparisons but remained exploratory. Across four breast cancer trials, pooled PFS was HR 0.65 (95% CI 0.54–0.79; *p* = 0.006; I^2^ = 13.4%), and pooled PFS in pathway-altered breast cancer populations was HR 0.53 (95% CI 0.34–0.82; *p* = 0.019; I^2^ = 41.5%). Across seven randomized trials reporting PFS, radiographic PFS, or composite PFS, the pooled HR was 0.73 (95% CI 0.62–0.85; *p* = 0.003; I^2^ = 50.6%). Across five randomized trials with mature or prespecified OS estimates, the pooled HR was 0.77 (95% CI 0.62–0.96; *p* = 0.030; I^2^ = 24.0%). Detailed results are provided in Supplementary Table S8.

### 3.10. Certainty of Evidence

The certainty of evidence was high for overall PFS and ORR with capivasertib plus fulvestrant in HR-positive/HER2-negative advanced breast cancer and for overall PFS with capivasertib plus paclitaxel in triple-negative breast cancer. Certainty was moderate for PFS in the *PIK3CA*/*AKT1*/*PTEN*-altered HR-positive/HER2-negative population, OS with fulvestrant, and radiographic PFS and interim OS in *PTEN*-deficient metastatic hormone-sensitive prostate cancer. Certainty was low for overall OS in triple-negative breast cancer, grade ≥ 3 and serious adverse events, and severe diarrhoea, rash, and hyperglycaemia. Certainty was very low for pathway-altered PFS and OS in triple-negative breast cancer, mCRPC outcomes, any-grade adverse events, treatment discontinuation, and adverse-event-related death. Downgrading reflected inconsistency, indirectness, risk of bias in exploratory analyses, and imprecision from immature data, sparse events, or wide confidence intervals, as shown in Table 4.

## 4. Discussion

This systematic review and meta-analysis indicates that the clinical effects of capivasertib vary according to tumor type, disease state, biomarker selection, and treatment backbone rather than reflecting a uniform effect of AKT inhibition. The most consistent randomized evidence was observed with capivasertib plus fulvestrant in HR-positive/HER2-negative advanced breast cancer, whereas findings in triple-negative breast cancer and prostate cancer varied according to clinical setting. These efficacy findings were accompanied by increased average risks of grade ≥ 3 toxicity and treatment discontinuation, although the magnitude of safety effects varied materially across studies. The noncomparative evidence demonstrated preliminary antitumor activity across several combinations but did not permit causal attribution or reliable comparisons between treatment partners. The clinical meaning of the pooled relative effects varied by endpoint. HRs for PFS or radiographic PFS reflect the relative risk of progression or death over follow-up but do not convey the absolute magnitude of benefit, whereas OS provides a more definitive measure of survival benefit but remained immature in several trials. ORR reflects the proportion of patients achieving tumor shrinkage and should be interpreted separately from time-to-event outcomes because it does not capture response duration or subsequent disease control [22]. Consequently, effect estimates across these endpoints should be interpreted within their specific clinical context rather than compared as equivalent measures of treatment benefit.

The strongest and most reproducible efficacy evidence concerned capivasertib plus fulvestrant in HR-positive/HER2-negative advanced breast cancer. FAKTION initially demonstrated improvements in PFS and OS after aromatase-inhibitor resistance, and CAPItello-291 subsequently confirmed the PFS benefit in a substantially larger phase III population [12,13]. CAPItello-291 is particularly relevant to current practice because approximately 69% of its participants had previously received a CDK4/6 inhibitor, whereas earlier endocrine-resistance trials generally included fewer patients exposed to this treatment class [13]. By contrast, BEECH did not demonstrate improved progression-free survival when capivasertib was combined with paclitaxel in ER-positive/HER2-negative disease, suggesting that biological activity cannot be generalized across combination backbones [11]. Although cross-trial comparisons cannot determine the optimal sequence, the relevant post-endocrine-treatment landscape now includes alpelisib plus fulvestrant for *PIK3CA*-mutated disease, inavolisib plus palbociclib and fulvestrant for endocrine-resistant *PIK3CA*-mutated disease, everolimus plus exemestane, elacestrant for ESR1-mutated disease, and trastuzumab deruxtecan for eligible HER2-low or HER2-ultralow disease [40,41,42,43,44,45,46]. These therapies address distinct resistance mechanisms and biomarker-defined populations and were evaluated in different treatment lines and combinations; they therefore should not be ranked or treated as interchangeable on the basis of indirect comparisons of relative effect estimates. Treatment selection should instead integrate the actionable molecular alteration, prior endocrine and CDK4/6-inhibitor exposure, HER2 expression, disease tempo, visceral involvement, metabolic comorbidity, and the feasibility of continuing an endocrine-based strategy.

The numerically stronger pooled PFS estimate in *PIK3CA*/*AKT1*/*PTEN*-altered breast cancer supports substantial activity in molecularly selected disease but does not establish predictive effect modification. The altered populations were contained within the overall trial populations, and no valid pooled interaction comparing altered and confirmed nonaltered tumors was available. FAKTION also showed that expanded molecular testing reclassified some tumors originally considered pathway nonaltered and that the treatment association was concentrated in the expanded pathway-altered population [12]. Consistent with this observation, the regulatory review of CAPItello-291 found a favorable benefit–risk profile in patients with qualifying *PIK3CA*, *AKT1*, or *PTEN* alterations but not in the biomarker-negative population [24]. The biomarker categories are not analytically interchangeable: activating *PIK3CA* and *AKT1* variants are genomic events, whereas *PTEN* deficiency may be defined by genomic loss-of-function or by loss of protein expression. CAPItello-291 used a qualifying genomic alteration framework linked to an FDA-approved tissue next-generation-sequencing companion diagnostic, whereas CAPItello-281 selected prostate tumors according to *PTEN* protein deficiency by immunohistochemistry [13,18,21]. PlasmaMATCH additionally demonstrated ctDNA-directed enrolment for *AKT1*-mutated breast cancer, while early capivasertib–fulvestrant cohorts showed activity in *AKT1E17K*-mutant and *PTEN*-mutant disease [8,9,28]. Differences in analyte, assay platform, specimen source, and positivity definition can therefore classify nonidentical patient populations and limit direct cross-trial biomarker comparisons [12,13,18,21,25]. Future randomised studies should report alteration-specific effects, assay platform, specimen source, tissue–plasma concordance, clonality, and the timing of biomarker sampling.

The triple-negative breast cancer findings illustrate the limitations of extrapolating small phase II signals to definitive clinical practice. PAKT reported favorable progression-free- and overall-survival signals, with a larger estimated effect in the pathway-altered subgroup; however, that subgroup contained only 28 patients [14]. CAPItello-290 subsequently confirmed a PFS association but did not meet its prespecified overall-survival endpoints in either the overall or pathway-altered population [15]. In the pooled pathway-altered analyses, the confidence intervals for both PFS and OS crossed 1 and heterogeneity was substantial; point estimates below 1 should therefore not be interpreted as evidence of a biomarker-specific treatment effect. A similar development pattern occurred with ipatasertib: LOTUS produced a favorable randomized phase II PFS signal, whereas the biomarker-selected phase III IPATunity130 trial did not confirm a significant benefit in *PIK3CA*/*AKT1*/*PTEN*-altered triple-negative breast cancer [47,48]. In BEGONIA, adding capivasertib to durvalumab plus paclitaxel also did not produce a substantial incremental benefit over the immunotherapy–taxane backbone [36]. Together, these findings suggest that the broad *PIK3CA*/*AKT1*/*PTEN* classification is insufficient to identify AKT-dependent triple-negative tumours. Potential explanations include the molecular heterogeneity of triple-negative breast cancer, variable *PTEN* assessment, subclonal pathway alterations, competing immune or cell-cycle drivers, and differences in taxane and capivasertib scheduling. Therefore, the current evidence demonstrates biological activity and progression delay but does not establish a routine clinical role for capivasertib in triple-negative breast cancer.

The prostate-cancer evidence further supports the importance of disease state, biomarker definition, and treatment partner. Reciprocal feedback between androgen–receptor and PI3K/AKT signalling provides a biological rationale for dual pathway inhibition in *PTEN*-deficient prostate cancer [4]. CAPItello-281 demonstrated improved radiographic progression-free survival when capivasertib was added to abiraterone, corticosteroid, and androgen-deprivation therapy in prospectively selected *PTEN*-deficient metastatic hormone-sensitive disease [18]. Conversely, RE-AKT and ProCAID enrolled later-line mCRPC populations and did not demonstrate statistically significant improvements in their primary efficacy outcomes [16,17]. The phase I RE-AKT programme additionally showed that enzalutamide reduced capivasertib exposure, providing a pharmacokinetic explanation that may have contributed to the limited phase II efficacy of this combination [29]. The AKT-inhibitor class experience is concordant: IPATential150 demonstrated a radiographic PFS benefit with ipatasertib plus abiraterone in *PTEN*-loss mCRPC disease, but the final analysis did not demonstrate a corresponding OS benefit [49,50]. These data suggest that prospective *PTEN*-protein selection, intervention before extensive castration resistance, and the use of an abiraterone backbone may be important determinants of benefit. The exploratory pooled prostate-cancer estimates from this review should remain secondary to the trial-specific findings because they combined hormone-sensitive and mCRPC disease, different partners, and nonidentical progression endpoints.

The safety findings demonstrate that capivasertib can increase treatment burden, but the magnitude of that burden is setting-dependent. Grade ≥ 3 adverse events and treatment discontinuation were increased on average, while the estimates for serious adverse events and adverse events with an outcome of death remained imprecise and should not be interpreted as evidence of equivalence. The 95% prediction intervals for any-grade adverse events (0.89–1.27) and grade ≥ 3 adverse events (0.59–4.67) crossed the null, reinforcing that the pooled RRs are average effects across clinically heterogeneous treatment settings rather than uniform toxicity multipliers. Differences in disease setting, treatment backbone, concomitant therapy, capivasertib dose and schedule, follow-up, patient selection, and adverse-event ascertainment are plausible contributors to this variability [11,12,13,14,15,16,17,18,22]. The excess toxicity was concentrated in diarrhoea, rash, and hyperglycaemia, consistent with the established pharmacological profile of AKT inhibition. Grade ≥ 3 events are clinically consequential, whereas dose interruption and dose reduction are management strategies that may permit treatment continuation but also indicate treatment burden; permanent discontinuation represents failure to maintain the assigned regimen. In CAPItello-291, frequent diarrhoea, rash, and hyperglycaemia generally developed early and were managed with supportive medication, temporary interruption, or dose reduction, although adverse events still led to permanent capivasertib discontinuation in a clinically relevant proportion of patients [25]. Patient-reported analyses from CAPItello-291 showed preservation of global health status and overall quality of life but greater deterioration in diarrhoea-related symptoms, indicating that stable global quality-of-life scores do not imply an absence of treatment burden [51]. Alpelisib plus fulvestrant provides a relevant pathway-targeted comparison because SOLAR-1 demonstrated a high incidence of clinically important hyperglycaemia, although the adverse-event profiles of PI3Kα and AKT inhibition differ and have not been compared directly [40]. In practice, capivasertib treatment requires baseline assessment of glucose regulation, early metabolic surveillance, prompt antidiarrhoeal therapy, proactive management of cutaneous toxicity, review of drug interactions, and timely dose interruption or reduction.

The noncomparative evidence supports continued development of selected capivasertib combinations but cannot identify a superior partner. The pooled objective response and clinical-benefit estimates confirm antitumour activity, but the significant difference in objective response according to partner class represents an ecological comparison across small cohorts with different tumour types, biomarker profiles, previous treatments, and response-evaluable denominators. For example, the high response rate observed with trastuzumab deruxtecan plus capivasertib in DESTINY-Breast08 cannot be attributed solely to AKT inhibition because trastuzumab deruxtecan has substantial independent efficacy in HER2-low and HER2-ultralow MBC [33,44]. Similarly, ComPAKT demonstrated the feasibility and preliminary activity of capivasertib plus olaparib in both BRCA1/2-mutant and non-BRCA1/2-mutant cancers, but its single-arm design could not establish whether AKT inhibition restored or extended PARP-inhibitor sensitivity [10]. The absence of a substantial incremental signal with capivasertib in the BEGONIA immunotherapy–taxane arm further illustrates why single-arm response rates require confirmation against an appropriate concurrent control [36]. These studies are most useful for selecting biologically plausible combinations, doses, and schedules for randomized evaluation rather than for defining comparative efficacy.

This review has several methodological strengths. Companion publications, updated survival reports, regional subgroups, and overlapping cohorts were linked to their parent trials to reduce double counting. Randomized and noncomparative evidence were analyzed separately, and the principal meta-analyses were restricted to disease- and backbone-specific comparisons. Outcome-specific risk-of-bias assessments were used rather than numerical quality scores, and the risk-of-bias judgments were incorporated into interpretation and GRADE certainty assessments. Sensitivity analyses confirmed the stability of the principal PFS findings; low-risk-only pooling was not forced when exclusion of a study with some concerns would leave only a single trial. Prostate-cancer outcomes, early-phase dose selection, pharmacokinetic interactions, and toxicity were retained even when clinical heterogeneity prevented definitive pooling.

Several limitations should also guide interpretation. Each principal comparative synthesis contained only two trials, limiting the precision of between-study variance estimates and preventing a reliable assessment of small-study effects. Aggregate data precluded patient-level interaction analyses according to individual genomic alteration, *PTEN* protein expression, previous CDK4/6-inhibitor exposure, visceral disease, metabolic risk, treatment adherence, or dose intensity. Clinical heterogeneity arose from differences in tumor type and subtype, disease state, treatment line and backbone, molecular-selection strategy, prior therapy, follow-up maturity, and endpoint definition across the included programmes. Biomarker methods differed across tumor types and clinical programmes, and overall-survival data remained immature or inconsistently reported in several trials. The exploratory analyses combined different cancers, disease states, treatment partners, and progression definitions and should not be interpreted as tumor-agnostic estimates. Safety definitions also varied regarding grade ≥ 3 versus grade 3–4 events, treatment-related versus all-cause events, and discontinuation of capivasertib versus discontinuation of any component. The wide prediction intervals for several heterogeneous outcomes further indicate that average pooled effects may not reproduce in every clinical setting. Finally, the treatment-line and study-phase subgroup analyses were underpowered study-level comparisons; their nonsignificant interaction tests do not demonstrate the absence of effect modification.

In conclusion, the randomized evidence indicates that capivasertib-containing regimens have setting-specific rather than tumor-agnostic effects. The most consistent comparative benefit was observed with capivasertib plus fulvestrant in HR-positive/HER2-negative advanced breast cancer, including the pathway-altered population. In triple-negative breast cancer, PFS favored capivasertib plus paclitaxel, but OS and biomarker-defined estimates remained inconclusive; in prostate cancer, outcomes differed between hormone-sensitive and mCRPC settings. Capivasertib was associated with higher average risks of grade ≥ 3 toxicity and treatment discontinuation, but substantial heterogeneity and wide prediction intervals indicate that the magnitude of toxicity varies across regimens and populations. Findings from uncontrolled prospective phase II and phase I/Ib cohorts should be regarded as hypothesis-generating evidence for activity, dose selection, safety, and pharmacokinetics rather than evidence of comparative efficacy. Evidence remains insufficient to support routine capivasertib use in triple-negative breast cancer, later-line mCRPC, or other solid tumors outside clinical trials. Research priorities include alteration-specific randomized analyses, harmonized genomic and immunohistochemical *PTEN* definitions, prospective tissue–ctDNA concordance, serial assessment of acquired resistance, and randomized evaluation of promising partners. Trials should also standardize adverse-event definitions, dose intensity, metabolic monitoring, and patient-reported outcomes so that incremental antitumor benefit can be evaluated against the full clinical burden of treatment.

## Figures and Tables

**Table 1:** Study design, treatment, and key patient characteristics of randomised comparative studies.

Study	Phase/Design	Disease Setting	Molecular Selection	Treatment Line	Experimental vs. Control Regimen	N (E/C)	Capivasertib Dose/Schedule	Median Age, y (E/C)	ECOG 0, n/N (%) (E/C)	Key Prior Treatment	Primary Endpoint/ Follow-Up
CAPItello-291 global [1,2,3,4]	III; Randomised, double-blind, placebo-controlled, parallel-group	HR-positive/HER2-negative advanced or metastatic breast cancer	Unselected; dual-primary altered subgroup defined by qualifying PIK3CA/AKT1/PTEN alterations	Second or later endocrine-based treatment; no prior fulvestrant	Capivasertib + fulvestrant vs. Placebo + fulvestrant	355/353	400 mg orally twice daily; 4 days on/3 days off each week	59.0/58.0	224/355 (63.1%)/241/353 (68.3%)	CDK4/6 inhibitor: 69.0%; chemotherapy for advanced disease: 18.3%	Investigator-assessed PFS in overall and altered populations; Median PFS follow-up was approximately 13.0 months
CAPItello-291 extended Chinese cohort [5]	III extension; Randomised, double-blind, placebo-controlled protocol-matched extension	HR-positive/HER2-negative advanced breast cancer in China	Unselected; altered subgroup prespecified	Post-aromatase-inhibitor advanced disease	Capivasertib + fulvestrant vs. Placebo + fulvestrant	71/63	400 mg orally twice daily; 4 days on/3 days off	56.0/54.0	35/71 (49.3%)/31/63 (49.2%)	CDK4/6 inhibitor: 38.0%; chemotherapy for advanced disease: 33.8%	Investigator-assessed PFS; NR
CAPItello-281 [6]	III; Randomised, double-blind, placebo-controlled, parallel-group	PTEN-deficient metastatic hormone-sensitive prostate cancer	PTEN deficiency required by immunohistochemistry	First-line systemic intensification for mHSPC	Capivasertib + abiraterone + corticosteroid + ADT vs. Placebo+ abiraterone+ corticosteroid + ADT	507/505	400 mg orally twice daily; 4 days on/3 days off	67.0/68.0	329/507 (64.9%)/320/505 (63.4%)	First-line systemic intensification for mHSPC	Radiographic PFS; Median follow-up 42.2 months approximately
BEECH Part B [7]	II; Randomised, double-blind, placebo-controlled	ER-positive/HER2-negative advanced or metastatic breast cancer	Unselected; stratified by PIK3CA mutation	First chemotherapy in advanced setting	Capivasertib + paclitaxel vs. Placebo + paclitaxel	54/56	400 mg twice daily; 4 days on/3 days off	53 (32–73)/60 (36–83)	WHO 0: 39/54 (72.2%)/42/56 (75.0%)	Prior taxane: 19/54 (35.2%)/22/56 (39.3%); prior hormonal therapy: 43/54 (79.6%)/39/56 (69.6%); median prior anticancer regimens: 2/2	PFS in overall and PIK3CA-mutant populations; NR
CAPItello-290 [8]	III; Randomised, double-blind, placebo-controlled	Previously untreated locally advanced or metastatic triple-negative breast cancer	Unselected; altered subgroup by central PIK3CA/AKT1/PTEN testing	First-line metastatic TNBC	Capivasertib + paclitaxel vs. Placebo + paclitaxel	404/408	400 mg twice daily; Days 2–5, weeks 1–3 of each 4-week cycle	54.0/53.0	236/404 (58.4%)/236/408 (57.8%)	First-line metastatic TNBC	OS in overall and altered populations; Median 16.6 vs. 16.8 months at final OS
RE-AKT phase II [9]	II; Randomised, double-blind, placebo-controlled	mCRPC after docetaxel and abiraterone	Unselected; PTEN status prespecified/exploratory	Later-line mCRPC	Capivasertib + enzalutamide vs. Placebo + enzalutamide	50/50	400 mg twice daily; 4 days on/3 days off	72.3/71.5	NR/NR	Later-line mCRPC	Composite response at 12 weeks; Median 43 months
PAKT [10]	II; Randomised, double-blind, placebo-controlled	Untreated metastatic triple-negative breast cancer	Unselected; PIK3CA/AKT1/PTEN subgroup	First-line metastatic TNBC	Capivasertib + paclitaxel vs. Placebo + paclitaxel	70/70	400 mg twice daily; 4 days on/3 days off	55.5/51.9	43/70 (61.4%)/48/70 (68.6%)	taxane: 57.1%	PFS; Median 18.2 months approximately
FAKTION [11]	II; Randomised, double-blind, placebo-controlled	Aromatase-inhibitor-resistant ER-positive/HER2-negative advanced breast cancer	Unselected; expanded pathway-altered subgroup	Endocrine-resistant advanced disease	Capivasertib + fulvestrant vs. Placebo + fulvestrant	69/71	400 mg twice daily; 4 days on/3 days off	62.0/61.0	42/69 (60.9%)/49/71 (69.0%)	chemotherapy for advanced disease: 24.6%	PFS; Median 58.5 months updated
ProCAID phase II [12,13]	II; Randomised, double-blind, placebo-controlled	Metastatic castration-resistant prostate cancer	Unselected; PI3K/AKT/PTEN biomarkers exploratory	Docetaxel-treated mCRPC	Capivasertib + docetaxel + prednisolone vs. Placebo + docetaxel + prednisolone	75/75	320 mg twice daily; 4 days on/3 days off	69.0/70.0	44/75 (58.7%)/46/75 (61.3%)	Docetaxel-treated mCRPC	Composite PFS; Updated OS follow-up reported in 2022

Values are reported as experimental/control unless otherwise stated. ECOG, Eastern Cooperative Oncology Group; E, experimental; C, control; HR, hormone receptor; HER2, human epidermal growth factor receptor 2; mCRPC, metastatic castration-resistant prostate cancer; mHSPC, metastatic hormone-sensitive prostate cancer; PFS, progression-free survival; RP2D, recommended phase II dose; TNBC, triple-negative breast cancer.

**Table 2:** Uncontrolled prospective phase II and phase I/Ib cohorts.

Study/Cohort	Phase/Design	Population and Molecular Selection	Combination Regimen	N	Age, Years	Performance Status 0, n/N (%)	Key Disease Characteristics	Key Prior Treatment
plasmaMATCH cohort C (NCT03182634) [1]	IIa; Prospective multicenter nonrandomized platform cohort	AKT1-mutated ER-positive advanced breast cancer	Capivasertib 400 mg twice daily, 4 days on/3 days off + fulvestrant	18	<50: 3/18 (16.7%); 50–59: 7/18 (38.9%); 60–69: 6/18 (33.3%); ≥70: 2/18 (11.1%)	NR at cohort level	De novo metastatic disease 5/18 (27.8%); visceral 17/18 (94.4%); bone 18/18 (100%)	Chemotherapy 15/18 (83.3%); endocrine therapy 18/18 (100%); CDK4/6 inhibitor 6/18 (33.3%)
BEECH Part A (NCT01625286) [2]	Ib; Open-label dose-finding safety run-in	Advanced breast cancer	Capivasertib + weekly paclitaxel; two intermittent capivasertib schedules	38 (Schedule 1, n = 20; Schedule 2, n = 18)	53 (35–76)/51 (31–70), median (range)	WHO 0: 11/20 (55.0%)/13/18 (72.2%)	WHO performance status 1: 9/20 (45.0%)/5/18 (27.8%)	Chemotherapy 18/20 (90.0%)/18/18 (100%); taxane 15/20 (75.0%)/9/18 (50.0%)
RE-AKT phase I (NCT02525068) [3]	I; Open-label dose-escalation	Metastatic castration-resistant prostate cancer	Capivasertib + enzalutamide	16 enrolled; 15 treated	70.4 (IQR 68.0–72.6), median	ECOG 0: 2/16 (12.5%)	Bone involvement 13/16 (81.3%); Gleason score ≥ 8, 9/16 (56.3%)	Docetaxel 16/16 (100%); abiraterone 14/16 (87.5%); enzalutamide 8/16 (50.0%); cabazitaxel 8/16 (50.0%)
ProCAID phase I (NCT02121639) [4]	I; Open-label 3 + 3 dose-escalation	Metastatic castration-resistant prostate cancer	Capivasertib + docetaxel + prednisolone	10	67.5 (56–72), median (range)	ECOG 0: 7/10 (70.0%)	Bone involvement 9/10 (90.0%); Gleason score 8–10, 5/10 (50.0%)	Prior abiraterone or enzalutamide 3/10 (30.0%)
DESTINY-Breast08 capivasertib module (NCT04556773) [5]	Ib; Open-label modular dose expansion	HER2-low metastatic breast cancer	Trastuzumab deruxtecan + capivasertib	40	56 (33–78), median (range)	ECOG 0: 30/40 (75.0%)	HR-negative 39/40 (97.5%); HER2 IHC 1+ 29/40 (72.5%)	No prior metastatic line 22/40 (55.0%); one prior line 18/40 (45.0%)
AKT1E17K fulvestrant combination cohort (NCT01226316) [6]	I; Open-label expansion cohort	AKT1E17K-mutant ER-positive/HER2-negative metastatic breast cancer	Capivasertib + fulvestrant	43 (fulvestrant-naive, n = 15; pretreated, n = 28)	58 (42–76)/56 (40–73), median (range)	WHO/ECOG 0: 4/15 (26.7%)/10/28 (35.7%)	Visceral disease 12/15 (80.0%)/23/28 (82.1%)	Prior metastatic chemotherapy 12/15 (80.0%)/26/28 (92.9%); prior CDK4/6 inhibitor 1/15 (6.7%)/11/28 (39.3%)
PTEN-mutant fulvestrant cohort (NCT01226316) [7]	I; Open-label expansion cohort	PTEN-mutant ER-positive metastatic breast cancer	Capivasertib + fulvestrant	31 treated (fulvestrant-naive, n = 12; pretreated, n = 19)	51 (31–68)/55 (42–70), mean (range)	WHO 0: 5/12 (41.7%)/8/19 (42.1%)	Visceral disease 12/12 (100%)/16/19 (84.2%)	Prior regimens median 7/7; CDK4/6 inhibitor 1/12 (8.3%)/12/19 (63.2%)
SERENA-1 parts I/J (NCT03616587) [8]	I; Open-label dose escalation and expansion	ER-positive/HER2-negative advanced breast cancer	Camizestrant 75 mg + capivasertib 400 mg twice daily, 4 days on/3 days off	29	53.0 (39–82), median (range)	ECOG 0: 13/29 (44.8%)	Measurable disease 27/29 (93.1%); visceral metastases 21/29 (72.4%)	Prior advanced regimens median 2 (1–6); CDK4/6 inhibitor 26/29 (89.7%); fulvestrant 16/29 (55.2%)
Chinese phase I PK study Part B (NCT04742036) [9]	I; Open-label fixed-sequence pharmacokinetic study	Chinese patients with advanced solid tumors	Capivasertib + paclitaxel combination period	16	55.5 (42–72), median (range)	ECOG 0: 0/16; ECOG 1: 16/16 (100%)	Breast primary 13/16 (81.3%); all had metastatic disease	Chemotherapy 16/16 (100%); hormonal therapy 11/16 (68.8%); targeted therapy 10/16 (62.5%)
BEGONIA capivasertib arm (NCT03742102) [10]	Ib; Open-label multiarm platform cohort	Untreated unresectable locally advanced or metastatic triple-negative breast cancer	Durvalumab + paclitaxel + capivasertib	31	51.0 (32–71), median (range)	ECOG 0: 17/31 (54.8%)	Visceral metastases 19/31 (61.3%); lymph-node metastases 19/31 (61.3%)	No prior treatment 14/31 (45.2%); prior taxane 13/31 (41.9%)
ComPAKT (NCT02338622) [11]	Ib; Open-label dose escalation and expansion	Previously treated advanced solid tumors	Olaparib + capivasertib using 4-days-on/3-days-off or 2-days-on/5-days-off schedules	64 (4/3 schedule, n = 33; 2/5 schedule, n = 31)	57 (32–76), median (range), overall	ECOG 0: 22/64 (34.4%)	Ovarian cancer 25/64 (39.1%); breast cancer 18/64 (28.1%)	Prior systemic lines median 4 (1–12); BRCA1/2-mutant cancer 25/64 (39.1%)
Olaparib-capivasertib dose expansion (NCT02208375) [12]	Ib; Single-center safety lead-in and dose expansion	Recurrent ovarian, endometrial, or triple-negative breast cancer	Olaparib + capivasertib	38	61.0 (30–75), median (range)	ECOG 0: 28/38 (73.7%)	Ovarian 16/38 (42.1%); endometrial 11/38 (28.9%); TNBC 11/38 (28.9%)	Prior treatment lines median 3 (1–7); germline BRCA1/2 mutation 7/38 (18.4%)
Capivasertib-abiraterone phase Ib (NCT04087174) [13]	Ib; Open-label multicenter two-part study	Metastatic castration-resistant prostate cancer	Capivasertib + abiraterone + prednisone/prednisolone	27	68 (49–82), median (range)	ECOG 0: 12/27 (44.4%)	Time from diagnosis 6.4 years (0.1–17.1); screening PSA 83.4 microg/L (0.40–593)	Docetaxel 22/27 (81.5%); enzalutamide 18/27 (66.7%); abiraterone 11/27 (40.7%); immunotherapy 13/27 (48.1%)
OLAPCO capivasertib cohort (NCT02576444) [14]	II; Open-label multiarm basket cohort	Molecularly selected treatment-refractory advanced solid tumors	Olaparib + capivasertib	16	NR at eligible-cohort level	NR at eligible-cohort level	Breast 4/16 (25.0%); cholangiocarcinoma 3/16 (18.8%); uterine 2/16 (12.5%); colorectal/rectal 2/16 (12.5%)	Cohort-specific prior-treatment distribution NR; population described as treatment refractory

These uncontrolled prospective cohorts are presented descriptively and were not combined with randomised comparative effects. Performance status is reported using the scale used in each source (ECOG or WHO). Slash-separated values correspond to the named subgroups or dosing schedules. NR indicates not reported for the eligible cohort. DLT, dose-limiting toxicity; ECOG, Eastern Cooperative Oncology Group; MTD, maximum tolerated dose; PK, pharmacokinetics; RP2D, recommended phase II dose; WHO, World Health Organization.

**Table 3:** Pooled Risk Ratios for Specific Adverse Events: Capivasertib vs. Control.

Adverse Event	Grade	No. of Studies	Pooled RR	95% CI	*p*-Value	I^2^
Diarrhoea	Any grade	6	2.78	1.51–5.11	0.008	94.8%
	Grade ≥3	8	8.21	3.59–18.79	<0.001	40.8%
Rash	Any grade	6	3.61	2.41–5.40	<0.001	68.8%
	Grade ≥3	6	23.47	9.10–60.56	<0.001	0.0%
Hyperglycaemia	Any grade	5	2.87	2.10–3.91	<0.001	22.2%
	Grade ≥3	6	12.30	7.06–21.42	<0.001	0.0%
Fatigue	Any grade	5	1.22	0.87–1.72	0.178	63.3%
	Grade ≥3	6	1.04	0.42–2.59	0.919	0.0%
Neutropenia	Any grade	3	1.20	0.68–2.10	0.301	0.0%
	Grade ≥3	4	1.19	0.98–1.44	0.065	0.0%
Nausea	Any grade	3	1.59	0.66–3.85	0.153	78.7%
	Grade ≥3	3	2.30	0.79–6.72	0.079	0.0%
Anaemia	Any grade	2	1.53	1.05–2.22	0.026	79.9%
	Grade ≥3	2	1.98	0.32–12.25	0.461	90.1%
Infection	Any grade	2	1.84	1.17–2.89	0.008	0.0%
	Grade ≥3	2	2.38	0.63–9.04	0.204	0.0%
Hypertension	Any grade	2	0.96	0.77–1.19	0.686	65.5%
	Grade ≥3	2	0.98	0.55–1.73	0.940	61.2%

RR, risk ratio; CI, confidence interval; I^2^, heterogeneity statistic. Random-effects models (REML) with Hartung–Knapp adjustment (k ≥ 3) or Wald CIs (k = 2).

**Table 4:** GRADE certainty of evidence for the principal efficacy and comparative safety outcomes of capivasertib-containing regimens.

Outcome and Comparison	Studies (Participants)	Relative Effect (95% CI)	Anticipated Absolute Effect *	Risk of Bias	Inconsistency	Indirectness	Imprecision	Publication Bias	Certainty of Evidence
**HR-positive/HER2-negative advanced breast cancer**									
PFS, overall population: capivasertib plus fulvestrant versus placebo plus fulvestrant	2 (848)	HR 0.59 (0.51–0.69)	Not estimated †	Not serious	Not serious	Not serious	Not serious	Not strongly suspected ^l^	⨁⨁⨁⨁ **High**
PFS, *PIK3CA*/*AKT1*/*PTEN*-altered population	2 (365)	HR 0.49 (0.38–0.62)	Not estimated †	Not serious	Not serious	Serious ^a^	Not serious	Not strongly suspected ^l^	⨁⨁⨁◯ **Moderate**
Objective response rate, overall population	2 (770)	RR 2.23 (1.31–3.80)	115 per 1000 with control and 257 per 1000 (151–437) with capivasertib; 142 more per 1000 (36–322 more)	Not serious	Not serious	Not serious	Not serious	Not strongly suspected ^l^	⨁⨁⨁⨁ **High**
Overall survival, overall population; one mature and one interim estimate	2 (848)	HR 0.71 (0.57–0.89)	Not estimated †	Not serious	Not serious	Not serious	Serious ^b^	Not strongly suspected ^l^	⨁⨁⨁◯ **Moderate**
**First-line advanced or metastatic triple-negative breast cancer**									
PFS, overall population: capivasertib plus paclitaxel versus placebo plus paclitaxel	2 (952)	HR 0.72 (0.62–0.84)	Not estimated †	Not serious	Not serious	Not serious	Not serious	Not strongly suspected ^l^	⨁⨁⨁⨁ **High**
Overall survival, overall population	2 (952)	HR 0.80 (0.55–1.17)	Not estimated †	Not serious	Serious ^c^	Not serious	Serious ^d^	Not strongly suspected ^l^	⨁⨁◯◯ **Low**
PFS, *PIK3CA*/*AKT1*/*PTEN*-altered population	2 (277)	HR 0.52 (0.24–1.15)	Not estimated †	Serious ^e^	Serious ^c^	Not serious	Serious ^d^	Not strongly suspected ^l^	⨁◯◯◯ **Very low**
Overall survival, *PIK3CA*/*AKT1*/*PTEN*-altered population	2 (277)	HR 0.72 (0.27–1.92)	Not estimated †	Serious ^e^	Serious ^c^	Not serious	Very serious ^f^	Not strongly suspected ^l^	⨁◯◯◯ **Very low**
**Prostate cancer**									
Radiographic PFS, PTEN-deficient metastatic hormone-sensitive prostate cancer: capivasertib plus abiraterone, corticosteroid, and ADT versus control	1 (1012)	HR 0.81 (0.66–0.98)	Not estimated †	Not serious	Not applicable	Not serious	Serious ^g^	Not strongly suspected ^l^	⨁⨁⨁◯ **Moderate**
Overall survival, PTEN-deficient metastatic hormone-sensitive prostate cancer; interim analysis	1 (1012)	HR 0.90 (0.71–1.15)	Not estimated †	Not serious	Not applicable	Not serious	Serious ^b^	Not strongly suspected ^l^	⨁⨁⨁◯ **Moderate**
Progression outcomes in metastatic castration-resistant prostate cancer; exploratory synthesis	2 (250)	HR 0.85 (0.70–1.03)	Not estimated †	Serious ^h^	Not serious	Serious ^i^	Serious ^d^	Not strongly suspected ^l^	⨁◯◯◯ **Very low**
Overall survival in metastatic castration-resistant prostate cancer	2 (250)	HR 0.73 (0.43–1.23)	Not estimated †	Serious ^h^	Not serious	Serious ^i^	Very serious ^f^	Not strongly suspected ^l^	⨁◯◯◯ **Very low**
**Comparative safety across randomised studies**									
Any-grade adverse event	5 (2806)	RR 1.06 (0.99–1.15)	909 per 1000 with control and 963 per 1000 (900–1000) with capivasertib; 55 more per 1000 (9 fewer to 91 more)	Not serious	Serious ^c^	Serious ^j^	Serious ^d^	Not strongly suspected ^l^	⨁◯◯◯ **Very low**
Grade ≥3 adverse event	5 (2778)	RR 1.66 (1.06–2.60)	332 per 1000 with control and 552 per 1000 (352–864) with capivasertib; 219 more per 1000 (20–532 more)	Not serious	Serious ^c^	Serious ^j^	Not serious	Not strongly suspected ^l^	⨁⨁◯◯ **Low**
Serious adverse event	2 (1815)	RR 1.59 (0.96–2.65)	220 per 1000 with control and 349 per 1000 (211–582) with capivasertib; 130 more per 1000 (9 fewer to 363 more)	Not serious	Not serious	Serious ^j^	Serious ^d^	Not strongly suspected ^l^	⨁⨁◯◯ **Low**
Adverse event with an outcome of death	2 (1815)	RR 1.41 (0.95–2.09)	44 per 1000 with control and 62 per 1000 (42–92) with capivasertib; 18 more per 1000 (2 fewer to 48 more)	Not serious	Not serious	Serious ^j^	Very serious ^f^	Not strongly suspected ^l^	⨁◯◯◯ **Very low**
Adverse event leading to treatment discontinuation	4 (2668)	RR 2.97 (1.27–6.96)	45 per 1000 with control and 134 per 1000 (57–313) with capivasertib; 89 more per 1000 (12–268 more)	Not serious	Serious ^c^	Serious ^j^	Serious ^d^	Not strongly suspected ^l^	⨁◯◯◯ **Very low**
Severe diarrhoea, defined as grade ≥3	8 (3154)	RR 8.21 (3.59–18.79)	9 per 1000 with control and 78 per 1000 (34–178) with capivasertib; 68 more per 1000 (25–169 more)	Not serious	Not serious	Serious ^j^	Serious ^k^	Not strongly suspected ^l^	⨁⨁◯◯ **Low**
Severe rash, defined as grade ≥3	6 (2908)	RR 23.47 (9.10–60.56)	2 per 1000 with control and 48 per 1000 (19–125) with capivasertib; 46 more per 1000 (17–123 more)	Not serious	Not serious	Serious ^j^	Serious ^k^	Not strongly suspected ^l^	⨁⨁◯◯ **Low**
Severe hyperglycaemia, defined as grade ≥3	6 (2908)	RR 12.30 (7.06–21.42)	3 per 1000 with control and 42 per 1000 (24–74) with capivasertib; 39 more per 1000 (21–70 more)	Not serious	Not serious	Serious ^j^	Serious ^k^	Not strongly suspected ^l^	⨁⨁◯◯ **Low**

Randomized evidence started at high certainty and was downgraded for serious or very serious concerns across the GRADE domains of risk of bias, inconsistency, indirectness, imprecision, and publication bias. * Anticipated absolute effects for binary outcomes were calculated by applying the pooled risk ratio to the pooled control-group event rate and were rounded to the nearest whole number per 1000 participants. † Absolute effects were not estimated for time-to-event outcomes because a common clinically relevant follow-up time and baseline event risk were not consistently available across contributing studies; hazard ratios were therefore reported without conversion to absolute survival differences. ADT, androgen-deprivation therapy; CI, confidence interval; GRADE, Grading of Recommendations Assessment, Development and Evaluation; HR, hazard ratio; OS, overall survival; PFS, progression-free survival; RR, risk ratio; TNBC, triple-negative breast cancer. ^a^ Downgraded one level for indirectness because biomarker ascertainment and the operational definition of PIK3CA/AKT1/PTEN-altered disease differed between the contributing trials. ^b^ Downgraded one level for imprecision because survival data were interim, immature, or incomplete and did not establish a precise magnitude of effect. ^c^ Downgraded one level for inconsistency because effect estimates differed materially between studies and/or statistical heterogeneity was substantial. ^d^ Downgraded one level for imprecision because the 95% CI crossed the null or included both little effect and a clinically important effect. ^e^ Downgraded one level for risk of bias because the biomarker-defined analyses were subgroup analyses with limited information and concerns regarding analysis or selective reporting. ^f^ Downgraded two levels for very serious imprecision because sparse information and wide 95% CIs included substantial benefit, no effect, and possible harm. ^g^ Downgraded one level for imprecision because evidence was provided by a single trial and the upper 95% confidence limit approached the null. ^h^ Downgraded one level for risk of bias because the contributing metastatic castration-resistant prostate-cancer trials had outcome-specific concerns related to composite endpoint measurement and selective reporting. ^i^ Downgraded one level for indirectness because the exploratory metastatic castration-resistant prostate-cancer synthesis combined different treatment backbones and nonidentical progression endpoints. ^j^ Downgraded one level for indirectness because comparative safety estimates were pooled across different tumour types and treatment backbones. ^k^ Downgraded one level for imprecision because events were uncommon and the 95% CIs remained wide despite a consistent direction of effect. ^l^ Formal assessment of publication bias or small-study effects was not possible because fewer than 10 studies contributed to each outcome; no downgrade was applied because no clear evidence of nonpublication was identified.

## Data Availability

No new data were created or analyzed in this study. Data sharing is not applicable to this article.

## Supplementary Materials

The following supporting information can be downloaded at: https://www.mdpi.com/article/10.3390/ph19101614/s1, Figure S1: Objective response rate across single-arm capivasertib combination cohorts, stratified by treatment partner; Figure S2: Clinical benefit rate across single-arm capivasertib combination cohorts, stratified by treatment partner; Figure S3: Proportion of patients experiencing grade ≥3 adverse events in single-arm capivasertib combination cohorts; Figure S4: Proportion of patients experiencing dose-limiting toxicity in phase I/Ib capivasertib combination cohorts; Figure S5: Progression-free survival with capivasertib-containing regimens according to treatment line; Figure S6: Progression-free survival with capivasertib-containing regimens according to study phase; Table S1: Full search strings by database; Table S2: Report-to-trial linkage, endpoint use, and overlap decisions; Table S3: Baseline patient and disease characteristics by study and treatment arm; Table S4: Treatment characteristics and dosing schedules of included capivasertib study arms; Table S5: Outcome-specific risk-of-bias assessment for randomized capivasertib trials using the Cochrane Risk of Bias 2 tool; Table S6: JBI critical appraisal of uncontrolled prospective phase I/Ib and single-arm phase II cohorts; Table S7: Sensitivity analyses of pooled efficacy outcomes ac-cording to statistical model and effect measure; Table S8: Exploratory pooled efficacy analyses across randomized capivasertib trials. References cited in the Supplementary Materials are included in the main reference list [8,9,10,11,12,13,14,15,16,17,18,24,25,26,27,28,29,30,31,32,33,34,35,36,37,38,39].
