# Supplementary material for: Capivasertib Combination Therapy in Advanced Breast Cancer and Other Solid Tumors: A Systematic Review and Meta-Analysis of Efficacy, Safety, and Biomarker-Defined Outcomes
Source: Pharmaceuticals (Basel). 2026 Oct 11;19(10):1614. doi: 10.3390/ph19101614 (PMC13708107; doi:10.3390/ph19101614)
Supplement: Supplementary file 1 [file pharmaceuticals-19-01614-s001.zip › Supplementary Figures Clean_revised .pdf]

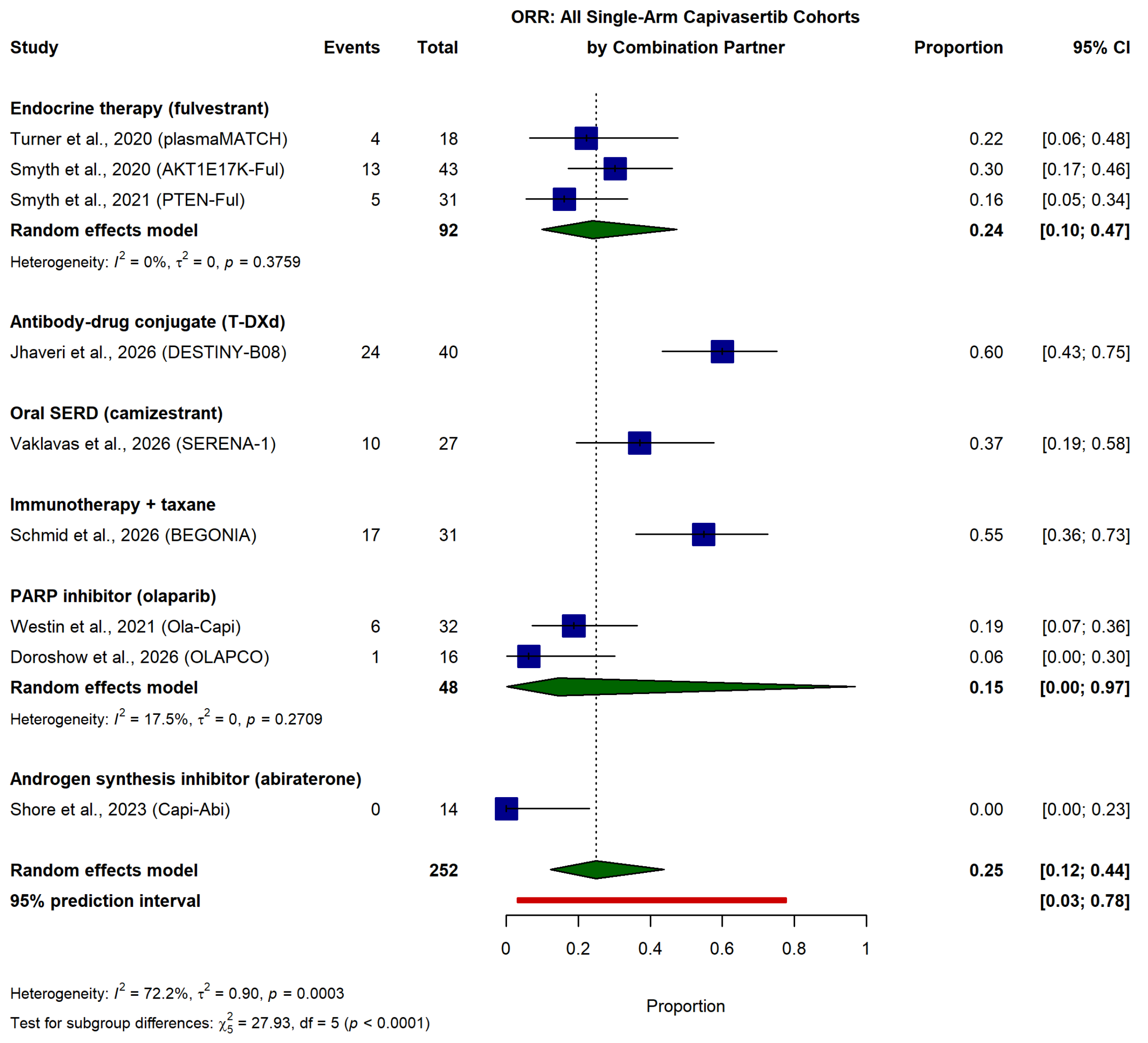

Figure S1. Objective response rate across single-arm capivasertib combination cohorts, stratified by treatment partner. Forest plot of objective response rates (ORRs) in single-arm phase II and phase I/Ib cohorts evaluating capivasertib-containing combinations. Study-specific proportions are shown with 95% confidence intervals (CIs), and diamonds represent random-effects pooled estimates. The red horizontal line represents the 95% prediction interval for the overall random-effects estimate. ORR, objective response rate; PARP, poly(ADP-ribose) polymerase; SERD, selective estrogen receptor degrader; T-DXd, trastuzumab deruxtecan.

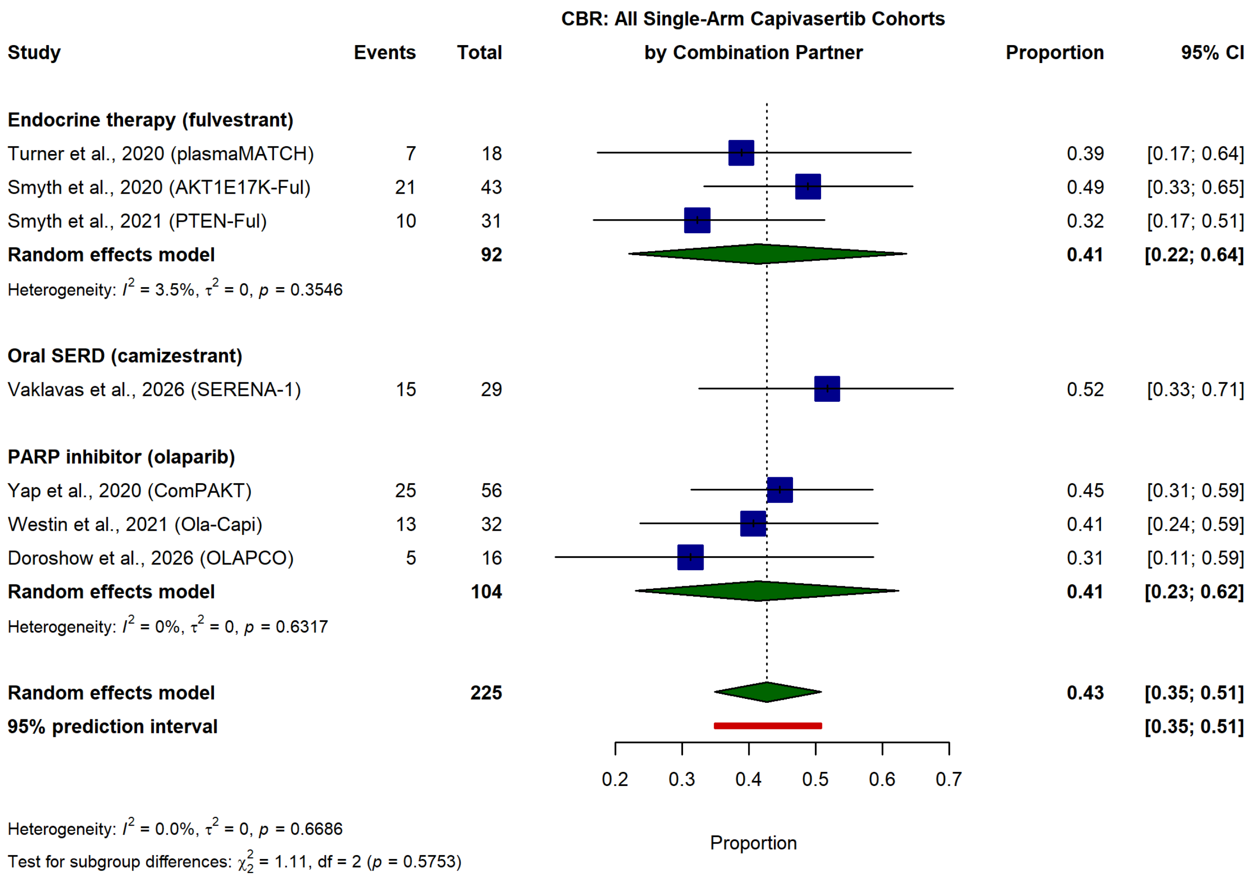

Figure S2. Clinical benefit rate across single-arm capivasertib combination cohorts, stratified by treatment partner. Forest plot of clinical benefit rates (CBRs) in single-arm capivasertib combination cohorts, stratified by endocrine therapy, oral selective estrogen receptor degrader, and poly(ADP-ribose) polymerase inhibitor partners. Study-specific proportions are presented with 95% confidence intervals (CIs), and diamonds indicate random-effects pooled estimates. The red horizontal line represents the 95% prediction interval for the overall random-effects estimate. CBR, clinical benefit rate; PARP, poly(ADP-ribose) polymerase; SERD, selective estrogen receptor degrader.

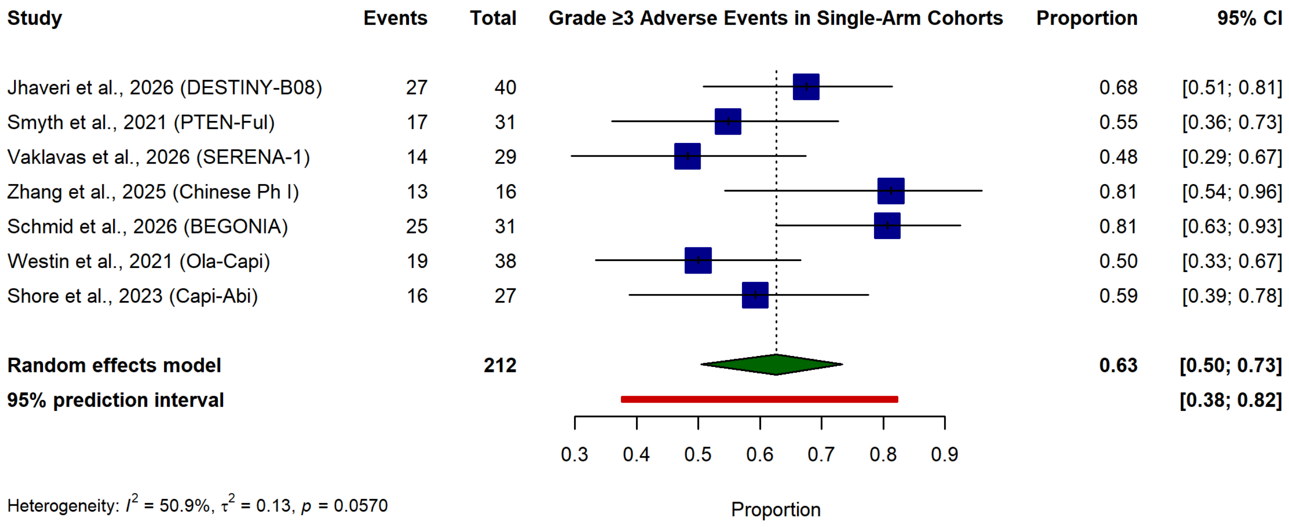

Figure S3. Proportion of patients experiencing grade ≥3 adverse events in single-arm capivasertib combination cohorts. Forest plot of grade ≥3 adverse-event proportions across single-arm phase II and phase I/Ib cohorts evaluating capivasertib-containing regimens. Study-specific proportions are shown with 95% confidence intervals (CIs), and the diamond represents the random-effects pooled estimate. The red horizontal line represents the 95% prediction interval for the overall random-effects estimate. AE, adverse event.

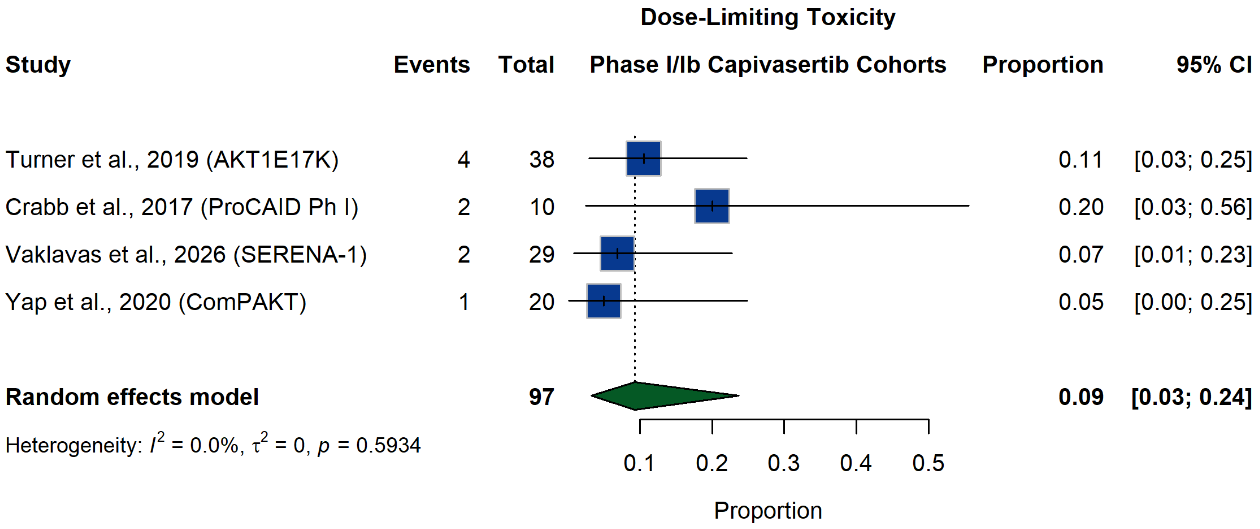

Figure S4. Proportion of patients experiencing dose-limiting toxicity in phase I/Ib capivasertib combination cohorts. Forest plot of dose-limiting toxicity (DLT) proportions across phase I and phase Ib capivasertib combination cohorts. Study-specific proportions are presented with 95% confidence intervals (CIs), and the diamond indicates the random-effects pooled estimate. DLT, dose-limiting toxicity.

Study	ln(HR)	SE[ln(HR)]	Capivasertib Total	Control Total	HR	95% CI	Weight
**Later-line**							
Turner et al., 2023 (CAPItello-291)	-0.5108	0.0844	355	353	0.60	[0.51; 0.71]	20.8%
Rescigno et al., 2024 (RE-AKT Ph II)	-0.2485	0.2595	50	50	0.78	[0.47; 1.30]	5.8%
Howell et al., 2022 (FAKTION)	-0.5798	0.1931	69	71	0.56	[0.38; 0.82]	9.0%
Crabb et al., 2021 (ProCAID Ph II)	-0.0834	0.1181	75	75	0.92	[0.73; 1.16]	16.0%
**Random effects model (HK)**					**0.70**	**[0.48; 1.02]**	**51.6%**
Heterogeneity: $I^2 = 70.1\%$, $\tau^2 = 0.04$, $p = 0.0183$							
**First-line**							
Fizazi et al., 2026 (CAPItello-281)	-0.2107	0.1008	507	505	0.81	[0.66; 0.99]	18.4%
Schmid et al., 2026 (CAPItello-290)	-0.3285	0.0816	404	408	0.72	[0.61; 0.84]	21.2%
Schmid et al., 2020 (PAKT)	-0.3011	0.1965	70	70	0.74	[0.50; 1.09]	8.8%
**Random effects model (HK)**					**0.75**	**[0.64; 0.89]**	**48.4%**
Heterogeneity: $I^2 = 0\%$, $\tau^2 = 0$, $p = 0.6594$							
**Random effects model (HK)**					**0.73**	**[0.62; 0.85]**	**100.0%**
**95% prediction interval**						**[0.51; 1.03]**	

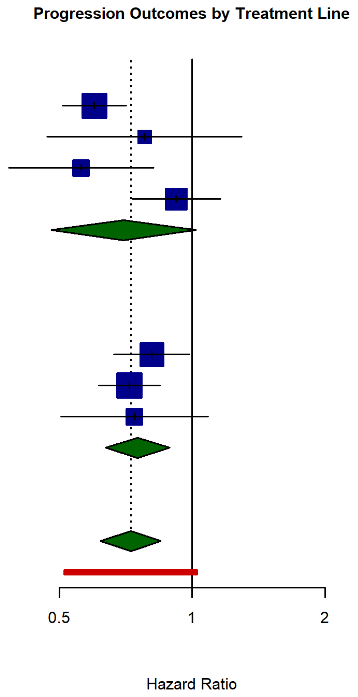

Heterogeneity: $I^2 = 50.6\%$, $\tau^2 = 0.02$, $p = 0.0589$

Test for subgroup differences: $\chi^2_1 = 0.35$, df = 1 ($p = 0.5532$)

Figure S5. Progression-free survival with capivasertib-containing regimens according to treatment line. Forest plot of progression outcomes from randomised studies stratified as first-line or later-line treatment. Hazard ratios (HRs) below 1 favour capivasertib plus the protocol-defined treatment backbone over the corresponding control regimen. The red horizontal line represents the 95% prediction interval for the overall random-effects estimate. Study-specific estimates are shown with 95% confidence intervals (CIs), with square size proportional to study weight; diamonds represent Hartung–Knapp random-effects pooled estimates.

Study	ln(HR)	SE[ln(HR)]	Capivasertib Total	Control Total	HR	95% CI	Weight
**Phase III**							
Turner et al., 2023 (CAPItello-291)	-0.5108	0.0844	355	353	0.60	[0.51; 0.71]	20.8%
Fizazi et al., 2026 (CAPItello-281)	-0.2107	0.1008	507	505	0.81	[0.66; 0.99]	18.4%
Schmid et al., 2026 (CAPItello-290)	-0.3285	0.0816	404	408	0.72	[0.61; 0.84]	21.2%
**Random effects model (HK)**					**0.70**	**[0.48; 1.02]**	**60.4%**
Heterogeneity: $I^2 = 63.8\%$, $\tau^2 = 0.01$, $p = 0.0631$							
**Phase II**							
Rescigno et al., 2024 (RE-AKT Ph II)	-0.2485	0.2595	50	50	0.78	[0.47; 1.30]	5.8%
Schmid et al., 2020 (PAKT)	-0.3011	0.1965	70	70	0.74	[0.50; 1.09]	8.8%
Howell et al., 2022 (FAKTION)	-0.5798	0.1931	69	71	0.56	[0.38; 0.82]	9.0%
Crabb et al., 2021 (ProCAID Ph II)	-0.0834	0.1181	75	75	0.92	[0.73; 1.16]	16.0%
**Random effects model (HK)**					**0.76**	**[0.54; 1.08]**	**39.6%**
Heterogeneity: $I^2 = 39.4\%$, $\tau^2 = 0.02$, $p = 0.1754$							
**Random effects model (HK)**					**0.73**	**[0.62; 0.85]**	**100.0%**
**95% prediction interval**						**[0.51; 1.03]**	

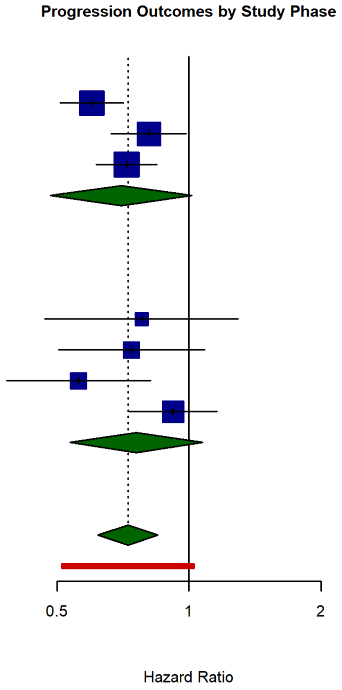

Heterogeneity: $I^2 = 50.6\%$, $\tau^2 = 0.02$, $p = 0.0589$

Test for subgroup differences: $\chi^2_1 = 0.33$, df = 1 ($p = 0.5660$)

Figure S6. Progression-free survival with capivasertib-containing regimens according to study phase. Forest plot of progression outcomes from randomised studies stratified as phase II or phase III. Hazard ratios (HRs) below 1 favour capivasertib plus the protocol-defined treatment backbone over the corresponding control regimen. The red horizontal line represents the 95% prediction interval for the overall random-effects estimate. Study-specific estimates are presented with 95% confidence intervals (CIs), with square size proportional to study weight; diamonds represent Hartung–Knapp random-effects pooled estimates.
