# Supplementary material for: Capivasertib Combination Therapy in Advanced Breast Cancer and Other Solid Tumors: A Systematic Review and Meta-Analysis of Efficacy, Safety, and Biomarker-Defined Outcomes
Source: Pharmaceuticals (Basel). 2026 Oct 11;19(10):1614. doi: 10.3390/ph19101614 (PMC13708107; doi:10.3390/ph19101614)
Supplement: Supplementary file 1 [file pharmaceuticals-19-01614-s001.zip › Supplementary Tables Clean_ Revised.pdf]

Supplementary Table S1. Full Search Strings by Database

Database	Full String	Records Retrieved
PubMed / MEDLINE	(Capivasertib[tiab]<br>OR AZD5363[tiab])<br>AND<br>(Neoplasms[mesh]<br>OR cancer*[tiab]<br>OR tumor*[tiab]<br>OR tumour*[tiab]<br>OR malignant*[tiab]<br>OR carcinoma*[tiab]<br>OR oncology[tiab]<br>OR "solid tumor*"[tiab]<br>OR "solid tumour*"[tiab]<br>OR "breast cancer"[tiab]<br>OR "breast neoplasm*"[tiab]<br>OR metastatic[tiab]<br>OR advanced[tiab])	319
WOS	TS=((Capivasertib OR AZD5363)<br>AND<br>TS=(cancer* OR tumor* OR tumour* OR malignant*<br>OR "solid tumor*" OR "solid tumour*"<br>OR "breast cancer"<br>OR metastatic OR advanced)	523
Scopus	TITLE-ABS-KEY<br>(Capivasertib OR AZD5363)<br>AND<br>TITLE-ABS-KEY<br>(cancer*<br>OR tumor*<br>OR tumour*<br>OR malignant*<br>OR "solid tumor*"<br>OR "breast cancer"<br>OR metastatic<br>OR advanced)	894
Cochrane CENTRAL	(Capivasertib OR AZD5363)<br>AND<br>(cancer* OR tumor* OR malignant*<br>OR "solid tumor*" OR "breast cancer")	127

Supplementary Table S2. Report-to-trial linkage, endpoint use, and overlap decisions

Study ID	Citation	Report role	Preferred endpoint use	Overlap/deduplication decision
CAPItello-291 global	Turner et al., 2023 [1]	Primary efficacy report	PFS; interim OS; global efficacy; principal safety	Parent global CAPItello-291 cohort (n=708)
	Dilawari et al., 2024 [2]	Regulatory companion	ORR; DOR; regulatory subgroup definitions	Complete overlap with global CAPItello-291
	Rugo et al., 2024 [3]	Detailed safety companion	Specific AEs; time to onset; dose modification; discontinuation	Complete overlap with global CAPItello-291
	Tokunaga et al., 2025 [4]	Geographic subgroup companion	Japan subgroup PFS; ORR; safety	78 participants nested within global CAPItello-291
CAPItello-291 extended Chinese cohort	Hu et al., 2025 [5]	Extension cohort primary report	Extension-only PFS; combined China efficacy/safety for descriptive use	Combined n=134 includes 24 global-trial participants and 110 additional extension participants
CAPItello-281	Fizazi et al., 2026 [6]	Primary efficacy and safety report	rPFS; interim OS; safety	Independent trial
plasmaMATCH cohort C	Turner et al., 2020 [7]	Platform-trial primary report	Cohort C ORR; PFS; safety	Only cohort C eligible; cohort D was monotherapy
BEECH Part A and B	Turner et al., 2019 [8]	Primary report for two distinct cohorts	Part A dose/safety; Part B PFS and safety	Part A and Part B are distinct cohorts within one report
CAPItello-290	Schmid et al., 2026 [9]	Primary efficacy and safety report	Final OS; PFS; safety	Independent trial
RE-AKT phase I	Kolinsky et al., 2020 [10]	Phase I dose-escalation report	RP2D; DLT; early safety/activity	Distinct from subsequent RE-AKT phase II cohort
RE-AKT phase II	Rescigno et al., 2024 [11]	Primary randomised report	Composite response; PFS; OS; safety	Independent phase II cohort
PAKT trial	Schmid et al., 2020 [12]	Primary randomised report	PFS; OS; response; safety	Independent trial
FAKTION trial	Jones et al., 2020 [13]	Original randomised report	Original methods; safety; initial PFS	Same 140 participants as Howell 2022

FAKTION trial	Howell et al., 2022 [14]	Updated follow-up report	Mature OS; updated PFS; expanded biomarker subgroups	Complete overlap with Jones 2020
ProCAID phase I	Crabb et al., 2017 [15]	Phase I dose-escalation report	RP2D; DLT; preliminary PSA response	Distinct precursor cohort
ProCAID phase II	Crabb et al., 2021 [16]	Primary randomised report	Composite PFS; response; safety; initial OS	Same phase II cohort as OS update
ProCAID phase II	Crabb et al., 2022 [17]	Follow-up companion	Updated OS	Complete overlap with phase II primary report
DESTINY-Breast08 capivasertib module	Jhaveri et al., 2026 [18]	Phase Ib module report	ORR; PFS; DOR; safety; ILD	Independent capivasertib module
AKT1E17K fulvestrant combination cohort	Smyth et al., 2020 [19]	Phase I expansion report	Combination cohort ORR; CBR24; PFS; safety	Combination cohort distinct from monotherapy cohort
PTEN-mutant fulvestrant cohort	Smyth et al., 2021 [20]	Phase I expansion report	ORR; CBR24; PFS; safety	Distinct PTEN-mutant cohort
SERENA-1 parts I/J	Vaklavas et al., 2026 [21]	Phase I combination report	PFS; CBR24; ORR; safety	Independent cohort
Chinese phase I PK study Part B	Zhang et al., 2025 [22]	Fixed-sequence phase I report	Combination-period PK; safety; exploratory response	Only combination period eligible
BEGONIA capivasertib arm	Schmid et al., 2026 [23]	Phase Ib platform-arm report	ORR; PFS; OS; DOR; safety	Independent part 1 arm; did not enter phase II expansion
ComPAKT	Yap et al., 2020 [24]	Phase Ib dose-escalation/expansion report	RP2D; ORR; CBR; safety; PK/PD	Independent trial
Olaparib–capivasertib dose expansion	Westin et al., 2021 [25]	Phase Ib expansion report	ORR; CBR; DOR; safety; translational outcomes	Independent from ComPAKT and OLAPCO
Capivasertib–abiraterone phase Ib	Shore et al., 2023 [26]	Phase Ib report	Safety; PK; PSA response; rPFS	Independent trial

OLAPCO capivasertib cohort	Doroshow et al., 2026 [27]	Phase II basket-cohort report	Capivasertib-cohort ORR; CBR; safety	Only cohort 3 eligible; other OLAPCO arms excluded

For studies reported in multiple publications, the parent or primary report was prioritised for principal efficacy and safety outcomes, while companion publications were used only for prespecified supplementary endpoints, updated follow-up, subgroup analyses, or detailed safety data. Completely overlapping reports were not treated as independent cohorts. Partially overlapping extension or geographic subgroup reports were retained for descriptive analyses only, with the overlapping participants explicitly identified to prevent double counting. Distinct cohorts reported within the same publication were assigned separate Study IDs when their participants, treatment settings, or study phases differed. DOI, digital object identifier; PFS, progression-free survival; OS, overall survival; rPFS, radiographic progression-free survival; ORR, objective response rate; DOR, duration of response; AE, adverse event; RP2D, recommended phase II dose; DLT, dose-limiting toxicity; CBR, clinical benefit rate; CBR24, clinical benefit rate at 24 weeks; PSA, prostate-specific antigen; PK, pharmacokinetics; PD, pharmacodynamics; ILD, interstitial lung disease.

Supplementary Table S3. Baseline patient and disease characteristics by study and treatment arm

Randomised comparative studies		
**CAPItello-291 global (NCT04305496; phase III): HR-positive/HER2-negative advanced or metastatic breast cancer**		
**Baseline characteristic**	**Capivasertib + fulvestrant (n=355)**	**Placebo + fulvestrant (n=353)**
Age	59 years (median)	58 years (median)
Female sex	352/355 (99.2%)	349/353 (98.9%)
ECOG performance status: 0	224/355 (63.1%)	241/353 (68.3%)
Visceral metastases	237/355 (66.8%)	241/353 (68.3%)
Liver metastases	156/355 (43.9%)	150/353 (42.5%)
Prior CDK4/6 inhibitor	245/355 (69.0%)	244/353 (69.1%)
Prior chemotherapy for advanced disease	65/355 (18.3%)	64/353 (18.1%)
PIK3CA/AKT1/PTEN-altered tumours (overall cohort)	289/708 (40.8%)	
**CAPItello-291 extended Chinese cohort (NCT04305496; phase III extension): HR-positive/HER2-negative advanced breast cancer in China**		
**Baseline characteristic**	**Capivasertib + fulvestrant (n=71)**	**Placebo + fulvestrant (n=63)**
Age	56 years (median)	54 years (median)
ECOG performance status: 0	35/71 (49.3%)	31/63 (49.2%)
Visceral metastases	52/71 (73.2%)	49/63 (77.8%)
Liver metastases	32/71 (45.1%)	29/63 (46.0%)
Prior CDK4/6 inhibitor	27/71 (38.0%)	23/63 (36.5%)
Prior chemotherapy for advanced disease	24/71 (33.8%)	16/63 (25.4%)
**CAPItello-281 (NCT04493853; phase III): PTEN-deficient metastatic hormone-sensitive prostate cancer**		
**Baseline characteristic**	**Capivasertib + abiraterone + corticosteroid + ADT (n=507)**	**Placebo + abiraterone + corticosteroid + ADT (n=505)**
Age	67 years (median)	68 years (median)
ECOG performance status: 0	329/507 (64.9%)	320/505 (63.4%)
Bone metastases	462/507 (91.1%)	467/505 (92.5%)

Liver metastases	30/507 (5.9%)	25/505 (5.0%)
High-risk disease	311/507 (61.3%)	333/505 (65.9%)
High-volume disease (overall cohort)	74.3%	
Gleason score ≥8 (overall cohort)	78.8%	
**BEECH Part B (NCT01625286; phase II): ER-positive/HER2-negative advanced or metastatic breast cancer**		
**Baseline characteristic**	**Capivasertib + paclitaxel (n=54)**	**Placebo + paclitaxel (n=56)**
Age, median (range), years	53 (32-73)	60 (36-83)
Race: White	24/54 (44.4%)	18/56 (32.1%)
Race: Asian	12/54 (22.2%)	15/56 (26.8%)
Race: American Indian or Alaska Native	15/54 (27.8%)	16/56 (28.6%)
Race: Other	3/54 (5.6%)	7/56 (12.5%)
WHO performance status: 0	39/54 (72.2%)	42/56 (75.0%)
WHO performance status: 1	15/54 (27.8%)	14/56 (25.0%)
PIK3CA mutation status: positive	26/54 (48.1%)	25/56 (44.6%)
PIK3CA mutation status: negative	28/54 (51.9%)	31/56 (55.4%)
Prior radiotherapy (any)	33/54 (61.1%)	33 (60%; denominator not stated)
Prior anticancer regimens, median (range)	2 (0-7)	2 (0-8)
Prior taxane use in any setting	19/54 (35.2%)	22/56 (39.3%)
Prior hormonal therapy in any setting	43/54 (79.6%)	39/56 (69.6%)
**CAPItello-290 (NCT03997123; phase III): Previously untreated locally advanced or metastatic triple-negative breast cancer**		
**Baseline characteristic**	**Capivasertib + paclitaxel (n=404)**	**Placebo + paclitaxel (n=408)**
Age	54 years (median)	53 years (median)
Female sex	404/404 (100.0%)	408/408 (100.0%)
ECOG performance status: 0	236/404 (58.4%)	236/408 (57.8%)
Visceral metastases	284/404 (70.3%)	284/408 (69.6%)
Liver metastases	96/404 (23.8%)	129/408 (31.6%)
Bone metastases	126/404 (31.2%)	133/408 (32.6%)
PD-L1 positive	58/404 (14.4%)	68/408 (16.7%)
PIK3CA/AKT1/PTEN-altered tumors (overall cohort)	249/812 (30.7%)	
**RE-AKT phase II (NCT02525068; phase II): mCRPC after docetaxel and abiraterone**		
**Baseline characteristic**	**Capivasertib + enzalutamide (n=50)**	**Placebo + enzalutamide (n=50)**

Age	72.3 years (median)	71.5 years (median)
White ethnicity	47/50 (94.0%)	50/50 (100.0%)
Metastatic disease at diagnosis	30/50 (60.0%)	29/50 (58.0%)
Measurable soft-tissue disease	31/50 (62.0%)	25/50 (50.0%)
Bone metastases	46/50 (92.0%)	47/50 (94.0%)
Liver metastases	7/50 (14.0%)	8/50 (16.0%)
PTEN normal	29/50 (58.0%)	33/50 (66.0%)
PTEN loss	16/50 (32.0%)	14/50 (28.0%)
**PAKT (NCT02423603; phase II): Untreated metastatic triple-negative breast cancer**		
**Baseline characteristic**	**Capivasertib + paclitaxel (n=70)**	**Placebo + paclitaxel (n=70)**
Age	55.5 years (median)	51.9 years (median)
ECOG performance status: 0	43/70 (61.4%)	48/70 (68.6%)
Visceral disease	42/70 (60.0%)	54/70 (77.1%)
Liver metastases	17/70 (24.3%)	21/70 (30.0%)
Lung metastases	35/70 (50.0%)	45/70 (64.3%)
Bone metastases	29/70 (41.4%)	28/70 (40.0%)
Prior taxane	40/70 (57.1%)	40/70 (57.1%)
PIK3CA/AKT1/PTEN-altered tumors (overall cohort)	28/140 (20.0%)	
**FAKTION (NCT01992952; phase II): Aromatase-inhibitor-resistant ER-positive/HER2-negative advanced breast cancer**		
**Baseline characteristic**	**Capivasertib + fulvestrant (n=69)**	**Placebo + fulvestrant (n=71)**
Age	62 years (median)	61 years (median)
ECOG performance status: 0	42/69 (60.9%)	49/71 (69.0%)
Visceral disease	49/69 (71.0%)	47/71 (66.2%)
Liver metastases	32/69 (46.4%)	29/71 (40.8%)
Bone metastases	58/69 (84.1%)	55/71 (77.5%)
Measurable disease	49/69 (71.0%)	50/71 (70.4%)
Prior chemotherapy for advanced disease	17/69 (24.6%)	20/71 (28.2%)
Prior endocrine lines in advanced setting	1 (median)	1 (median)
Expanded pathway-altered tumors (overall cohort)	76/140 (54.3%)	
**ProCAID phase II (NCT02121639; phase II): Metastatic castration-resistant prostate cancer**		

Baseline characteristic	Capivasertib + docetaxel + prednisolone (n=75)	Placebo + docetaxel + prednisolone (n=75)
Age	69 years (median)	70 years (median)
ECOG performance status: 0	44/75 (58.7%)	46/75 (61.3%)
Bone metastases	61/75 (81.3%)	63/75 (84.0%)
Visceral metastases	14/75 (18.7%)	17/75 (22.7%)
Prior abiraterone and/or enzalutamide	51/75 (68.0%)	50/75 (66.7%)
Gleason score 8–10	51/75 (68.0%)	40/75 (53.3%)
**Single-arm phase II and phase I/Ib cohorts**		
**plasmaMATCH cohort C (NCT03182634; phase IIa): AKT1-mutated ER-positive advanced breast cancer**		
**Baseline characteristic**	**Capivasertib + fulvestrant (n=18)**	
Age group: <50 years	3/18 (16.7%)	
Age group: 50-59 years	7/18 (38.9%)	
Age group: 60-69 years	6/18 (33.3%)	
Age group: ≥70 years	2/18 (11.1%)	
De novo metastatic disease	5/18 (27.8%)	
Histology: ductal	13/18 (72.2%)	
Histology: lobular	3/18 (16.7%)	
Histology: mixed ductal/lobular	1/18 (5.6%)	
HR-positive/HER2-negative	16/18 (88.9%)	
HR-positive/HER2-positive	1/18 (5.6%)	
HR-positive/HER2 unknown	1/18 (5.6%)	
Visceral metastases	17/18 (94.4%)	
Soft-tissue or nodal metastases	11/18 (61.1%)	
Bone metastases	18/18 (100%)	
Prior chemotherapy for advanced disease	15/18 (83.3%)	
Prior endocrine therapy for advanced disease	18/18 (100%)	
Prior mTOR inhibitor	3/18 (16.7%)	
Prior CDK4/6 inhibitor	6/18 (33.3%)	
Prior anti-HER2 therapy	1/18 (5.6%)	
**BEECH Part A (NCT01625286; phase Ib): Advanced breast cancer**		
**Baseline characteristic**	**Schedule 1 (n=20)**	**Schedule 2 (n=18)**

Age, median (range), years	53 (35-76)	51 (31-70)
White race	18/20 (90.0%)	18/18 (100%)
Asian race	1/20 (5.0%)	0/18 (0%)
Black or African American race	1/20 (5.0%)	0/18 (0%)
WHO performance status 0	11/20 (55.0%)	13/18 (72.2%)
WHO performance status 1	9/20 (45.0%)	5/18 (27.8%)
Prior chemotherapy	18/20 (90.0%)	18/18 (100%)
Prior taxane	15/20 (75.0%)	9/18 (50.0%)
Prior anticancer treatment for metastatic disease	6/20 (30.0%)	4/18 (22.2%)
**RE-AKT phase I (NCT02525068; phase I): Metastatic castration-resistant prostate cancer**		
Age, median (IQR), years	70.4 (68.0-72.6)	
Caucasian ethnicity	15/16 (93.8%)	
African-Caribbean ethnicity	1/16 (6.3%)	
ECOG performance status 0	2/16 (12.5%)	
ECOG performance status 1	14/16 (87.5%)	
Gleason score <8	4/16 (25.0%)	
Gleason score >=8	9/16 (56.3%)	
Gleason score unavailable	3/16 (18.8%)	
Metastatic disease at diagnosis	8/16 (50.0%)	
Metastatic sites: lymph nodes only	3/16 (18.8%)	
Metastatic sites: bone only	7/16 (43.8%)	
Metastatic sites: bone and lymph nodes	3/16 (18.8%)	
Metastatic sites: visceral and bone	2/16 (12.5%)	
Metastatic sites: visceral, bone, and lymph nodes	1/16 (6.3%)	
Prior abiraterone	14/16 (87.5%)	
Prior cabazitaxel	8/16 (50.0%)	
Prior docetaxel	16/16 (100%)	
Prior enzalutamide	8/16 (50.0%)	
**ProCAID phase I (NCT02121639; phase I): Metastatic castration-resistant prostate cancer**		
**Baseline characteristic**	**Capivasertib + docetaxel + prednisolone (n=10)**	
Age, median (range), years	67.5 (56-72)	

Age ≥65 years	7/10 (70.0%)	
ECOG performance status 0	7/10 (70.0%)	
ECOG performance status 1	3/10 (30.0%)	
Gleason score <=6	0/10 (0%)	
Gleason score 7	4/10 (40.0%)	
Gleason score 8-10	5/10 (50.0%)	
Metastatic sites: bone only	5/10 (50.0%)	
Metastatic sites: visceral only	1/10 (10.0%)	
Metastatic sites: visceral and bone	4/10 (40.0%)	
Prior abiraterone or enzalutamide	3/10 (30.0%)	
**DESTINY-Breast08 capivasertib module (NCT04556773; phase Ib): HER2-low metastatic breast cancer**		
**Baseline characteristic**	**Trastuzumab deruxtecan + capivasertib (n=40)**	
Age, median (range), years	56 (33-78)	
Female sex	40/40 (100%)	
Asian race	18/40 (45.0%)	
White race	18/40 (45.0%)	
Black or African American race	3/40 (7.5%)	
HER2 IHC 1+	29/40 (72.5%)	
HER2 IHC 2+/ISH-negative	10/40 (25.0%)	
HER2 IHC 2+/ISH missing	1/40 (2.5%)	
Hormone receptor-negative disease	39/40 (97.5%)	
ECOG performance status 0	30/40 (75.0%)	
ECOG performance status 1	10/40 (25.0%)	
No prior line for metastatic breast cancer	22/40 (55.0%)	
One prior line for metastatic breast cancer	18/40 (45.0%)	
PIK3CA/AKT1/PTEN-altered among evaluable	13/34 (38.2%)	
**AKT1E17K fulvestrant combination cohort (NCT01226316; phase I): AKT1E17K-mutant ER-positive metastatic breast cancer**		
**Baseline characteristic**	**Fulvestrant-naive (n=15)**	**Fulvestrant-pretreated (n=28)**
Age, median (range), years	58 (42-76)	56 (40-73)
Female sex	15/15 (100%)	28/28 (100%)
White race	9/15 (60.0%)	18/28 (64.3%)

Asian race	6/15 (40.0%)	5/28 (17.9%)
WHO/ECOG performance status 0	4/15 (26.7%)	10/28 (35.7%)
WHO/ECOG performance status 1	11/15 (73.3%)	18/28 (64.3%)
ER-positive/PR-positive	11/15 (73.3%)	23/28 (82.1%)
ER-positive/PR-negative	4/15 (26.7%)	5/28 (17.9%)
Visceral disease	12/15 (80.0%)	23/28 (82.1%)
Prior anticancer regimens, median (range)	4 (1-7)	6 (2-12)
Prior chemotherapy regimens, median (range)	2 (0-5)	2 (0-6)
Prior endocrine regimens, median (range)	1 (0-4)	4 (2-6)
Prior sensitivity to endocrine therapy	7/15 (46.7%)	16/28 (57.1%)
Prior chemotherapy for metastatic disease	12/15 (80.0%)	26/28 (92.9%)
Prior CDK4/6 inhibitor	1/15 (6.7%)	11/28 (39.3%)
Prior mTOR inhibitor	2/15 (13.3%)	9/28 (32.1%)
Prior PI3K inhibitor	1/15 (6.7%)	4/28 (14.3%)
Black race	NR	1/28 (3.6%)
Other/missing race	NR	4/28 (14.3%)
**PTEN-mutant fulvestrant cohort (NCT01226316; phase I): PTEN-mutant ER-positive metastatic breast cancer**		
**Baseline characteristic**	**Fulvestrant-naive (n=12)**	**Fulvestrant-pretreated (n=19)**
Age, mean (range), years	51 (31-68)	55 (42-70)
Female sex	12/12 (100%)	19/19 (100%)
White race	10/12 (83.3%)	16/19 (84.2%)
Black or African American race	1/12 (8.3%)	1/19 (5.3%)
Other race	1/12 (8.3%)	1/19 (5.3%)
WHO performance status 0	5/12 (41.7%)	8/19 (42.1%)
WHO performance status 1	7/12 (58.3%)	11/19 (57.9%)
Visceral disease	12/12 (100%)	16/19 (84.2%)
ER-positive/PR-positive	10/12 (83.3%)	17/19 (89.5%)
ER-positive/PR-negative	2/12 (16.7%)	1/19 (5.3%)
HER2-positive	1/12 (8.3%)	2/19 (10.5%)
Prior anticancer regimens, median (range)	7 (1-11)	7 (3-13)
Prior chemotherapy regimens, median (range)	4 (0-10)	2 (1-8)

Prior endocrine regimens, median (range)	2 (0-4)	4 (1-6)
Prior CDK4/6 inhibitor	1/12 (8.3%)	12/19 (63.2%)
Prior mTOR inhibitor	3/12 (25.0%)	9/19 (47.4%)
Prior PI3K inhibitor	0/12 (0%)	2/19 (10.5%)
Asian race	NR	1/19 (5.3%)
**SERENA-1 parts I/J (NCT03616587; phase I): ER-positive/HER2-negative advanced breast cancer**		
**Baseline characteristic**	**Camizestrant + capivasertib (n=29)**	
Age, median (range), years	53.0 (39-82)	
Postmenopausal	27/29 (93.1%)	
ECOG performance status 0	13/29 (44.8%)	
Measurable disease	27/29 (93.1%)	
Visceral metastases	21/29 (72.4%)	
Prior regimens in advanced setting, median (range)	2 (1-6)	
Prior endocrine regimens in advanced setting, median (range)	2 (0-5)	
Prior chemotherapy regimens in advanced setting, median (range)	0 (0-3)	
Prior fulvestrant in advanced setting	16/29 (55.2%)	
Prior CDK4/6 inhibitor in advanced setting	26/29 (89.7%)	
Baseline ESR1 mutation detected	17/29 (58.6%)	
Baseline PIK3CA/AKT1/PTEN alteration detected	15/29 (51.7%)	
**Chinese phase I PK study Part B (NCT04742036; phase I): Advanced solid tumors in Chinese patients**		
**Baseline characteristic**	**Capivasertib + paclitaxel (n=16)**	
Age, median (range), years	55.5 (42-72)	
Age <50 years	5/16 (31.3%)	
Age 50 to <65 years	9/16 (56.3%)	
Age 65 to <75 years	2/16 (12.5%)	
Female sex	14/16 (87.5%)	
Asian race	16/16 (100%)	
ECOG performance status 0	0/16 (0%)	
ECOG performance status 1	16/16 (100%)	
Primary tumor: breast	13/16 (81.3%)	
Primary tumor: nasopharynx	1/16 (6.3%)	

Primary tumor: prostate	1/16 (6.3%)	
Primary tumor: urethra	1/16 (6.3%)	
Prior cytotoxic chemotherapy	16/16 (100%)	
Prior hormonal therapy	11/16 (68.8%)	
Prior targeted therapy	10/16 (62.5%)	
Prior radiotherapy	7/16 (43.8%)	
Prior immunotherapy	3/16 (18.8%)	
**BEGONIA capivasertib arm (NCT03742102; phase Ib): Untreated unresectable locally advanced or metastatic TNBC**		
**Baseline characteristic**	**Durvalumab + paclitaxel + capivasertib (n=31)**	
Age, median (range), years	51.0 (32-71)	
Age <65 years	26/31 (83.9%)	
Age 65 to <75 years	5/31 (16.1%)	
Asian race	16/31 (51.6%)	
White race	15/31 (48.4%)	
No prior treatment	14/31 (45.2%)	
Prior radiotherapy	15/31 (48.4%)	
Prior cytotoxic chemotherapy	16/31 (51.6%)	
Prior anthracycline	15/31 (48.4%)	
Prior taxane	13/31 (41.9%)	
Prior hormonal therapy	2/31 (6.5%)	
Visceral metastases	19/31 (61.3%)	
Lymph-node metastases	19/31 (61.3%)	
ECOG performance status 0	17/31 (54.8%)	
ECOG performance status 1	14/31 (45.2%)	
PD-L1 TAP >=10%	4/31 (12.9%); 2 missing	
PIK3CA/AKT1/PTEN alteration	8/31 (25.8%); 3 missing	
**ComPAKT (NCT02338622; phase Ib): Previously treated advanced solid tumors**		
**Baseline characteristic**	**Olaparib + capivasertib (n=64)**	
Age, median (range), years	57 (32-76)	
Male sex	11/64 (17.2%)	
Female sex	53/64 (82.8%)	

ECOG performance status 0	22/64 (34.4%)	
ECOG performance status 1	42/64 (65.6%)	
Ovarian cancer	25/64 (39.1%)	
Breast cancer	18/64 (28.1%)	
Pancreatic cancer	5/64 (7.8%)	
mCRPC	4/64 (6.3%)	
Prior systemic lines, median (range)	4 (1-12)	
BRCA1/2-mutant cancer	25/64 (39.1%)	
**Olaparib–capivasertib dose expansion (NCT02208375; phase Ib): Recurrent endometrial, TNBC, ovarian, fallopian tube, or peritoneal cancer**		
**Baseline characteristic**	**Olaparib + capivasertib (n=38)**	
Age	61 years (median)	
Prior systemic lines	3 (median)	
Cancer type: Ovarian cancer	16/38 (42.1%)	
Cancer type: Endometrial cancer	11/38 (28.9%)	
Cancer type: Triple-negative breast cancer	11/38 (28.9%)	
Germline BRCA1/2 mutation	7/38 (18.4%)	
**Capivasertib–abiraterone phase Ib (NCT04087174; phase Ib): Metastatic castration-resistant prostate cancer**		
**Baseline characteristic**	**Capivasertib + abiraterone + prednisone/prednisolone (n=27)**	
Age, median (range), years	68 (49-82)	
Country: Spain	11/27 (40.7%)	
Country: USA	16/27 (59.3%)	
Time from diagnosis to enrollment, median (range), years	6.4 (0.1-17.1)	
ECOG performance status 0	12/27 (44.4%)	
ECOG performance status 1	15/27 (55.6%)	
PSA at screening, median (range), microg/L	83.4 (0.40-593)	
Prior abiraterone	11/27 (40.7%)	
Prior enzalutamide	18/27 (66.7%)	
Prior bicalutamide	9/27 (33.3%)	
Prior docetaxel	22/27 (81.5%)	
Prior cabazitaxel	9/27 (33.3%)	
Prior carboplatin	5/27 (18.5%)	

Prior immunotherapy	13/27 (48.1%)	
Prior PARP inhibitor	7/27 (25.9%)	
Prior radiotherapy	4/27 (14.8%)	
**OLAPCO capivasertib cohort (NCT02576444; phase II): Molecularly selected treatment-refractory advanced solid tumours**		
**Baseline characteristic**	**Olaparib + capivasertib (n=16)**	
Age	NR at cohort level	
Performance status	NR at cohort level	
Breast cancer	4/16 (25.0%)	
Cholangiocarcinoma	3/16 (18.8%)	
Uterine cancer	2/16 (12.5%)	
Colorectal/rectal cancer	2/16 (12.5%)	
NSCLC	1/16 (6.3%)	
Head and neck squamous cell carcinoma	1/16 (6.3%)	
Pancreatic cancer	1/16 (6.3%)	
Hepatocellular carcinoma	1/16 (6.3%)	
Adenoid cystic carcinoma	1/16 (6.3%)	
AKT1-mutated tumors	6/16 (37.5%)	
ARID1A-altered tumors	8/16 (50.0%)	
Cohort-specific prior-treatment distribution	NR	

Percentages were calculated using the reported arm-specific or evaluable denominator. Overall-cohort biomarker values are shown in merged cells. NR indicates that a value was not reported for the corresponding arm in the extracted dataset. ADT, androgen-deprivation therapy; BRCA1/2, breast cancer gene 1/2; CDK4/6, cyclin-dependent kinase 4/6; ECOG, Eastern Cooperative Oncology Group; ER, estrogen receptor; HER2, human epidermal growth factor receptor 2; HR, hormone receptor; mCRPC, metastatic castration-resistant prostate cancer; PARP, poly(ADP-ribose) polymerase; PD-L1, programmed death-ligand 1; SERD, selective estrogen receptor degrader; TNBC, triple-negative breast cancer.

Supplementary Table S4: Treatment characteristics and dosing schedules of included capivasertib study arms.

Study ID	Arm	Role	Assigned N	Treated N	Capivasertib dose	Schedule	Partner(s)	Cycle	Notes
S01	Capivasertib + fulvestrant	Experimental	355	355	400 mg orally twice daily	4 days on/3 days off each week	Fulvestrant 500 mg IM according to standard schedule	28 days	Dose reductions to 320 mg then 200 mg twice daily permitted.
S01	Placebo + fulvestrant	Control	353	350	Placebo	Matched intermittent schedule	Fulvestrant 500 mg IM	28 days	
S02	Capivasertib + fulvestrant	Experimental	71	71	400 mg orally twice daily	4 days on/3 days off	Fulvestrant	28 days	Combined China cohort; extension-only n not arm-separated in all outcomes.
S02	Placebo + fulvestrant	Control	63	62	Placebo	Matched	Fulvestrant	28 days	
S03	Capivasertib + abiraterone + corticosteroid + ADT	Experimental	507	503	400 mg orally twice daily	4 days on/3 days off	Abiraterone 1000 mg daily + prednisone/prednisolone + ADT	28 days	
S03	Placebo + abiraterone + corticosteroid + ADT	Control	505	503	Placebo	Matched	Abiraterone + corticosteroid + ADT	28 days	
S04	Capivasertib + fulvestrant	Single arm		18	400 mg twice daily	4 days on/3 days off	Fulvestrant	28 days	AKT1-mutated cohort C.
S05	Capivasertib + paclitaxel	Single arm dose-finding		38	Multiple schedules; selected 400 mg twice daily	Selected 4 days on/3 days off	Paclitaxel 90 mg/m² days 1, 8, 15	28 days	Part A.
S06	Capivasertib + paclitaxel	Experimental	54	54	400 mg twice daily	4 days on/3 days off	Paclitaxel 90 mg/m² days 1, 8, 15	28 days	
S06	Placebo + paclitaxel	Control	56	56	Placebo	Matched	Paclitaxel 90 mg/m²	28 days	
S07	Capivasertib + paclitaxel	Experimental	404	402	400 mg twice daily	Days 2–5, weeks 1–3 of each 4-week cycle	Paclitaxel 80 mg/m² day 1, weeks 1–3	28 days	
S07	Placebo + paclitaxel	Control	408	407	Placebo	Matched	Paclitaxel 80 mg/m²	28 days	
S08	Capivasertib + enzalutamide	Single arm dose-escalation		15	Dose escalation; RP2D 400 mg twice daily	4 days on/3 days off	Enzalutamide 160 mg daily	28 days	
S09	Capivasertib + enzalutamide	Experimental	50	48	400 mg twice daily	4 days on/3 days off	Enzalutamide 160 mg daily	28 days	Response-evaluable denominator 47; safety denominator 48.
S09	Placebo + enzalutamide	Control	50	50	Placebo	Matched	Enzalutamide 160 mg daily	28 days	Response-evaluable denominator 48; safety denominator 50.
S10	Capivasertib + paclitaxel	Experimental	70	68	400 mg twice daily	4 days on/3 days off	Paclitaxel 90 mg/m² days 1, 8, 15	28 days	

S10	Placebo + paclitaxel	Control	70	70	Placebo	Matched	Paclitaxel	28 days	
S11	Capivasertib + fulvestrant	Experimental	69	69	400 mg twice daily	4 days on/3 days off	Fulvestrant 500 mg IM	28 days	
S11	Placebo + fulvestrant	Control	71	71	Placebo	Matched	Fulvestrant	28 days	
S12	Capivasertib + docetaxel + prednisolone	Single arm dose-escalation		10	Dose escalation; RP2D 320 mg twice daily	4 days on/3 days off	Docetaxel + prednisolone	21 days/combination protocol	
S13	Capivasertib + docetaxel + prednisolone	Experimental	75	75	320 mg twice daily	4 days on/3 days off	Docetaxel 75 mg/m² q3w + prednisolone	21 days	Safety denominator reported as 74 in some tables; verify endpoint-specific denominator.
S13	Placebo + docetaxel + prednisolone	Control	75	75	Placebo	Matched	Docetaxel + prednisolone	21 days	
S14	Trastuzumab deruxtecan + capivasertib	Single arm module		40	400 mg twice daily	4 days on/3 days off	Trastuzumab deruxtecan 5.4 mg/kg IV every 3 weeks	21 days	
S15	Capivasertib + fulvestrant	Single arm biomarker cohort		43	400 mg twice daily	4 days on/3 days off	Fulvestrant	28 days	Safety denominator included one non-AKT1E17K participant in report.
S16	Capivasertib + fulvestrant	Single arm biomarker cohort		31	400 mg twice daily	4 days on/3 days off	Fulvestrant	28 days	
S17	Camizestrant + capivasertib	Single arm dose-escalation/expansion		29	Dose levels including 400 mg twice daily	4 days on/3 days off	Camizestrant oral SERD	28 days	
S18	Capivasertib + paclitaxel	Single arm combination period		16	400 mg twice daily	4 days on/3 days off	Paclitaxel	28 days	Fixed-sequence study; preceding monotherapy period excluded.
S19	Durvalumab + paclitaxel + capivasertib	Single arm platform cohort		31	400 mg twice daily	Days 2–5, 9–12, and 16–19	Durvalumab 1500 mg q4w + paclitaxel 80/90 mg/m²	28 days	
S20	Olaparib + capivasertib	Single arm dose-escalation/expansion		64	400 mg twice daily (4/3 RP2D) or 640 mg twice daily (2/5 RP2D)	4 days on/3 off or 2 days on/5 off	Olaparib 300 mg twice daily	28 days	
S21	Olaparib + capivasertib	Single arm safety lead-in/expansion		38	400 mg twice daily RP2D	4 days on/3 days off	Olaparib 300 mg twice daily	28 days	
S22	Capivasertib + abiraterone + prednisone/prednisolone	Single arm phase Ib		27	400 mg twice daily	4 days on/3 days off	Abiraterone 1000 mg daily + prednisone/prednisolone 10 mg/day	28 days	
S23	Olaparib + capivasertib	Single arm phase II cohort		16	640 mg twice daily	Days 1 and 2 of each week	Olaparib 300 mg twice daily continuously	28 days	

Study arms were summarized according to treatment role, numbers assigned and treated, capivasertib dose and schedule, combination partner, and cycle duration. Assigned N denotes the randomized or allocated population, whereas Treated N denotes participants who received at least one dose of study treatment. Blank Assigned N values indicate non-randomized dose-escalation, expansion, module, or single-arm cohorts for which only the treated population was applicable or reported. Where multiple capivasertib schedules were evaluated, the recommended phase II dose and schedule were identified. Endpoint-specific evaluable populations may differ from the treated population and are reported in the Notes column where relevant. ADT, androgen-deprivation therapy; IM, intramuscular; IV, intravenous; q3w, every 3 weeks; q4w, every 4 weeks; RP2D, recommended phase II dose; SERD, selective estrogen receptor degrader.

Supplementary Table S5. Outcome-specific risk-of-bias assessment for randomised capivasertib trials using the Cochrane Risk of Bias 2 tool.

Study ID	Outcome class	D1	D2	D3	D4	D5	Overall	Rationale
CAPItello-291 global	Time-to-event efficacy	Low risk	Low risk	Low risk	Low risk	Low risk	Low risk	Central randomisation and masking were used; ITT analyses were reported; missingness was limited; PFS was prospectively defined and investigator-assessed under masking; the primary analysis followed the prespecified hierarchy.
CAPItello-291 global	Safety	Low risk	Low risk	Low risk	Low risk	Low risk	Low risk	Blinded allocation, near-complete safety populations, standardised CTCAE collection, and detailed companion reporting were available.
CAPItello-291 extended Chinese cohort	Time-to-event efficacy	Low risk	Low risk	Low risk	Low risk	Some concerns	Some concerns	The randomised design was preserved, but extension-only reporting was secondary, and the published combined cohort overlapped the global trial; selective availability of extension-only endpoints limits certainty.
CAPItello-291 extended Chinese cohort	Safety	Low risk	Low risk	Low risk	Low risk	Some concerns	Some concerns	Safety ascertainment was standardised, but most safety estimates were reported for the combined Chinese cohort, which contained overlapping global participants.
CAPItello-281	Radiographic PFS/OS	Low risk	Low risk	Low risk	Low risk	Low risk	Low risk	Central randomised double-blind design, ITT analysis, protocol-defined imaging, and prespecified statistical testing were reported.
CAPItello-281	Safety	Low risk	Low risk	Low risk	Low risk	Low risk	Low risk	The blinded safety population was nearly complete and adverse events were collected using standardised criteria with detailed reporting.
BEECH Part B	PFS	Some concerns	Low risk	Low risk	Low risk	Some concerns	Some concerns	The study was randomised and double blinded, but the main report provided limited detail on sequence generation/concealment and used a small phase II design with 80% confidence intervals and multiple populations.
BEECH Part B	Safety	Some concerns	Low risk	Low risk	Low risk	Some concerns	Some concerns	Blinded safety assessment was used, although some detailed safety data were confined to supplementary material and randomisation-process details were limited in the main report.
CAPItello-290	OS/PFS	Low risk	Low risk	Low risk	Low risk	Low risk	Low risk	Large international randomised double-blind trial with ITT analyses, prespecified dual primary endpoints, mature OS, and standardised imaging.

CAPItello-290	Safety	Low risk	Low risk	Low risk	Low risk	Low risk	Low risk	Near-complete safety populations and detailed CTCAE-grade reporting were available under blinded allocation.
RE-AKT phase II	Composite response/PFS/OS	Low risk	Low risk	Some concerns	Some concerns	Some concerns	Some concerns	Randomisation and masking were reported, but five randomised patients were not evaluable for the composite primary response endpoint; the composite combined RECIST, PSA, and CTC components; several analyses were exploratory.
RE-AKT phase II	Safety	Low risk	Low risk	Low risk	Low risk	Some concerns	Some concerns	Safety populations were nearly complete, but detailed safety reporting was less comprehensive than efficacy reporting, and some results were reported selectively by event.
PAKT	PFS/OS/response	Low risk	Low risk	Low risk	Low risk	Some concerns	Some concerns	The trial was randomised and double blinded with ITT analyses; however, a one-sided phase II design, multiple secondary/subgroup analyses, and a small altered subgroup increase reporting-selection concerns.
PAKT	Safety	Low risk	Low risk	Low risk	Low risk	Low risk	Low risk	Safety denominators and grade-specific events were clearly reported for all treated participants.
FAKTION	PFS/OS/response	Low risk	Low risk	Low risk	Low risk	Low risk	Low risk	Randomised double-blind design, ITT analyses, mature follow-up, and transparent linkage of original and updated reports were available.
FAKTION	Safety	Low risk	Low risk	Low risk	Low risk	Some concerns	Some concerns	Safety data were detailed, but the small phase II sample and absence of a dedicated prespecified safety publication create minor reporting-selection concerns.
ProCAID phase II	Composite PFS/OS	Low risk	Low risk	Low risk	Some concerns	Some concerns	Some concerns	The randomised double-blind design was appropriate, but composite PFS was dominated by PSA progression and updated OS was a secondary endpoint reported after extended follow-up; interpretation therefore requires caution.
ProCAID phase II	Safety	Low risk	Low risk	Low risk	Low risk	Low risk	Low risk	Complete randomised safety populations, CTCAE grading, and arm-level event reporting were available.

Risk of bias was assessed separately for time-to-event efficacy, response-based efficacy, and safety outcomes because bias may differ across outcome classes within the same trial. Each outcome was evaluated across five domains: D1, bias arising from the randomisation process; D2, bias due to deviations from intended interventions; D3, bias due to missing outcome data; D4, bias in outcome measurement; and D5, bias in selection of the reported result. Domain-level judgments were classified as low risk, some concerns, or high risk, and the overall judgment was derived according to the Cochrane RoB 2 algorithm. For reports containing overlapping populations or updated follow-up, judgments reflected the specific dataset and outcome used in the synthesis. CTCAE, Common Terminology Criteria for Adverse Events; CTC, circulating tumour cells; ITT, intention-to-treat; OS, overall survival; PFS, progression-free survival; PSA, prostate-specific antigen; RECIST, Response Evaluation Criteria in Solid Tumours

Supplementary Table S6. JBI critical appraisal of uncontrolled prospective phase I/Ib and single-arm phase II cohorts

Study ID	Q1	Q2	Q3	Q4	Q5	Q6	Q7	Q8	Q9	Q10	Overall	Rationale
plasmaMATCH cohort C	Yes	Yes	Yes	Unclear	Yes	Yes	Yes	Yes	Yes	Yes	Moderate concern	Prospective biomarker-defined platform cohort with standardised response assessment; consecutive inclusion was not clearly documented, and the cohort was small.
BEECH Part A	Yes	Yes	Yes	Unclear	Yes	Yes	Yes	Yes	Yes	Yes	Moderate concern	Dose-finding cohort with clear methods and complete safety reporting; consecutive recruitment and some baseline details were confined to supplements.
RE-AKT phase I	Yes	Yes	Yes	Unclear	Yes	Yes	Yes	Yes	Yes	Yes	Moderate concern	Phase I dose-escalation cohort with standardised safety and response methods; selection into dose levels was nonconsecutive by design.
ProCAID phase I	Yes	Yes	Yes	Unclear	Yes	Yes	Yes	Yes	Yes	Yes	Moderate concern	Small 3+3 dose-escalation cohort with clear dose-limiting toxicity assessment; consecutive inclusion was not established.
DESTINY-Breast08 capivasertib module	Yes	Yes	Yes	Unclear	Yes	Yes	Yes	Yes	Yes	Yes	Moderate concern	Modular phase Ib cohort with standardised RECIST and safety assessment; slot availability and module selection limit certainty regarding consecutive inclusion.
AKT1E17K fulvestrant combination cohort	Yes	Yes	Yes	Unclear	Yes	Yes	Yes	Yes	Yes	Yes	Moderate concern	Molecularly defined expansion cohort with clear outcomes; the report combined fulvestrant-naive and pretreated strata, and the safety denominator included one additional participant.
PTEN-mutant fulvestrant cohort	Yes	Yes	Yes	Unclear	Yes	Yes	Yes	Yes	Yes	Yes	Moderate concern	Molecularly selected expansion cohort with complete outcome reporting; consecutive inclusion was not explicitly stated.
SERENA-1 parts I/J	Yes	Yes	Yes	Unclear	Yes	Yes	Yes	Yes	Yes	Yes	Moderate concern	Dose-escalation/expansion cohort with standardised efficacy and safety outcomes; selection into parts I/J was not clearly consecutive.
Chinese phase I PK study Part B	Yes	Yes	Yes	Unclear	Yes	Yes	Yes	Yes	Yes	Yes	Moderate concern	Fixed-sequence PK cohort with complete safety and response reporting; the small sample and specialised eligibility limit generalizability, and consecutive inclusion was unclear.
BEGONIA capivasertib arm	Yes	Yes	Yes	No	Yes	Yes	Yes	Yes	Yes	Yes	Moderate concern	Simon two-stage platform cohort with complete reporting, but enrollment into open novel arms was not consecutive and the capivasertib arm did not proceed to expansion.

ComPAKT	Yes	Yes	Yes	Unclear	Yes	Yes	Yes	Yes	Yes	Yes	Moderate concern	Multicenter phase Ib trial with clear RP2D, safety, and activity methods; biomarker-enriched expansion and dose-level allocation preclude confirmation of consecutive inclusion.
Olaparib–capivasertib dose expansion	Yes	Yes	Yes	Unclear	Yes	Yes	Yes	Yes	Yes	Yes	Moderate concern	Safety lead-in and expansion were clearly described with comprehensive translational reporting; slot-based enrollment and small tumour-specific subgroups limit certainty.
Capivasertib–abiraterone phase Ib	Yes	Yes	Yes	Unclear	Yes	Yes	Yes	No	Yes	Yes	High concern	All 27 treated patients contributed to safety, but only 14 were evaluable for objective tumour response; efficacy completeness was therefore limited and selection into evaluable analyses may bias activity estimates.
OLAPCO capivasertib cohort	Yes	Yes	Yes	Unclear	Yes	Yes	Yes	Yes	Yes	Yes	Moderate concern	Prospective Simon phase II basket cohort with complete participant-level outcomes; small heterogeneous tumour groups and unclear consecutive inclusion limit certainty.

Methodological quality of these uncontrolled prospective cohorts was assessed using the Joanna Briggs Institute Critical Appraisal Checklist for Case Series. The checklist was selected because these noncomparative early-phase and single-arm studies lacked a concurrent control group and its domains directly assess inclusion criteria, participant inclusion, standardised measurement, completeness of clinical and outcome reporting, study setting, and appropriateness of statistical analysis. The domains were: Q1, clear inclusion criteria; Q2, standardized and reliable measurement of the condition; Q3, valid identification methods; Q4, consecutive participant inclusion; Q5, complete participant inclusion; Q6, clear reporting of demographic characteristics; Q7, clear reporting of clinical information; Q8, complete reporting of outcomes and follow-up; Q9, clear reporting of the clinical setting; and Q10, appropriate statistical analysis. Responses were classified as Yes, No, Unclear, or Not applicable. Overall ratings of moderate or high concern were review-defined summary judgments based on the number and clinical importance of unresolved domains and were not numerical scores prescribed by JBI. JBI, Joanna Briggs Institute; PK, pharmacokinetics; RECIST, Response Evaluation Criteria in Solid Tumours; RP2D, recommended phase II dose.

Supplementary Table S7. Sensitivity analyses of pooled efficacy outcomes according to statistical model and effect measure

Analysis	Stratum	Outcome	Primary (RE)	Sensitivity	Interpretation
RE vs. FE	HR-positive/HER 2-negative	PFS	HR 0.59 [0.51, 0.69]	HR 0.59 [0.51, 0.69]	Identical (τ^2=0)
RE vs. FE	TNBC	PFS	HR 0.72 [0.62, 0.84]	HR 0.72 [0.62, 0.84]	Identical (τ^2=0)
RE vs. FE	TNBC	OS	HR 0.80 [0.55, 1.17]	HR 0.88 [0.76, 1.03]	Divergent; RE wider due to heterogeneity
RR vs. OR	HR-positive/HER 2-negative	ORR	RR 2.23 [1.31, 3.80]	OR 2.67 [1.39, 5.14]	Both significant
IV vs. MH	HR-positive/HER 2-negative	ORR	RR 2.23 [1.31, 3.80]	MH RR 2.09 [1.50, 2.90]	Consistent; MH slightly more precise

Primary analyses were conducted using random-effects models. Fixed-effect models were examined to assess the influence of between-study heterogeneity on pooled time-to-event outcomes. For objective response rate, odds ratios and Mantel–Haenszel pooling were evaluated as sensitivity analyses against the prespecified risk-ratio and inverse-variance approaches. Consistency was assessed according to the direction, magnitude, precision, and statistical significance of the pooled estimates. Risk-of-bias-restricted sensitivity analyses were also considered in accordance with Cochrane guidance. When exclusion of studies with some concerns left only one eligible comparison, no pooled sensitivity estimate was calculated; the remaining low-risk trial estimate was considered descriptively. CI, confidence interval; FE, fixed effect; HR, hazard ratio; IV, inverse variance; MH, Mantel–Haenszel; OR, odds ratio; ORR, objective response rate; OS, overall survival; PFS, progression-free survival; RE, random effects; RR, risk ratio; SAP, statistical analysis plan; τ^2, estimated between-study variance.

Supplementary Table S8. Exploratory pooled efficacy analyses across randomised capivasertib trials.

**Analysis**	**Outcome**	**Population**	**No. of Studies**	**Model**	**HR/RR**	**95% CI**	**p**	**I²**
All Breast Cancer RCTs	PFS	Overall	4	REML+HK	0.65	[0.54, 0.79]	0.006	13.4%
All Randomised Trials	PFS/rPFS/cPFS	Overall	7	REML+HK	0.73	[0.62, 0.85]	0.003	50.6%
All Randomised Trials	OS	Overall	5	REML+HK	0.77	[0.62, 0.96]	0.030	24.0%
All Breast Cancer (Altered)	PFS	Altered	4	REML+HK	0.53	[0.34, 0.82]	0.019	41.5%

Pooled hazard ratios were calculated for progression-free survival and overall survival, while effect estimates across progression-free survival definitions were synthesised only when considered clinically compatible. Random-effects models were fitted using restricted maximum likelihood estimation with Hartung–Knapp adjustment. Analyses included all breast cancer randomised controlled trials, all eligible randomised trials across tumour types, and the biomarker-altered breast cancer population. HR values below 1 favoured capivasertib-containing regimens. CI, confidence interval; cPFS, composite progression-free survival; HK, Hartung–Knapp adjustment; HR, hazard ratio; I^2, proportion of between-study variability attributable to heterogeneity; k, number of included comparisons; OS, overall survival; PFS, progression-free survival; RCT, randomized controlled trial; REML, restricted maximum likelihood; rPFS, radiographic progression-free survival; RR, risk ratio

References

1. Turner NC, Oliveira M, Howell SJ, Dalenc F, Cortes J, Gomez Moreno HL, et al. Capivasertib in Hormone Receptor–Positive Advanced Breast Cancer. N Engl J Med. 2023;388: 2058–2070. doi:10.1056/NEJMoa2214131

2. Dilawari A, Buturla J, Osgood C, Gao X, Chen W, Ricks TK, et al. US Food and Drug Administration Approval Summary: Capivasertib With Fulvestrant for Hormone Receptor–Positive, Human Epidermal Growth Factor Receptor 2–Negative Locally Advanced or Metastatic Breast Cancer With PIK3CA / AKT1 / PTEN Alterations. J Clin Oncol. 2024;42: 4103–4113. doi:10.1200/JCO.24.00427

3. Rugo HS, Oliveira M, Howell SJ, Dalenc F, Cortes J, Gomez HL, et al. Capivasertib and fulvestrant for patients with hormone receptor-positive advanced breast cancer: characterization, time course, and management of frequent adverse events from the phase III CAPItello-291 study. ESMO Open. 2024;9: 103697. doi:10.1016/j.esmoop.2024.103697

4. Tokunaga E, Iwata H, Itoh M, Taira T, Toyama T, Mizuno T, et al. Capivasertib and fulvestrant for patients with HR-positive/HER2-negative advanced breast cancer: analysis of the subgroup of patients from Japan in the phase 3 CAPItello-291 trial. Breast Cancer. 2025;32: 132–143. doi:10.1007/s12282-024-01640-z

5. Hu X, Zhang Q, Sun T, Xiong H, Li W, Teng Y, et al. Capivasertib plus fulvestrant in patients with HR-positive/HER2-negative advanced breast cancer: phase 3 CAPItello-291 study extended Chinese cohort. Nat Commun. 2025;16: 4324. doi:10.1038/s41467-025-59210-6

6. Fizazi K, Clarke NW, De Santis M, Uemura H, Fay AP, Karadurmus N, et al. Capivasertib plus abiraterone in PTEN-deficient metastatic hormone-sensitive prostate cancer: CAPItello-281 phase III study. Ann Oncol. 2026;37: 53–68. doi:10.1016/j.annonc.2025.10.004

7. Turner NC, Kingston B, Kilburn LS, Kernaghan S, Wardley AM, Macpherson IR, et al. Circulating tumour DNA analysis to direct therapy in advanced breast cancer (plasmaMATCH): a multicentre, multicohort, phase 2a, platform trial. Lancet Oncol. 2020;21: 1296–1308. doi:10.1016/S1470-2045(20)30444-7

8. Turner NC, Alarcón E, Armstrong AC, Philco M, López Chuken YA, Sablin M-P, et al. BEECH: a dose-finding run-in followed by a randomised phase II study assessing the efficacy of AKT inhibitor capivasertib (AZD5363) combined with paclitaxel in patients with estrogen receptor-positive advanced or metastatic breast cancer, and in a PIK3CA mutant sub-population. Ann Oncol Off J Eur Soc Med Oncol. 2019;30: 774–780. doi:10.1093/annonc/mdz086

9. Schmid P, McArthur HL, Cortés J, Xu B, Cardoso F, Casalnuovo M, et al. Capivasertib plus paclitaxel as first-line treatment for metastatic triple-negative breast cancer: results from the randomised, global phase III CAPItello-290 trial. Ann Oncol. 2026;37: 650–662.

doi:10.1016/j.annonc.2025.12.012

10. Kolinsky MP, Rescigno P, Bianchini D, Zafeiriou Z, Mehra N, Mateo J, et al. A phase I dose-escalation study of enzalutamide in combination with the AKT inhibitor AZD5363 (capivasertib) in patients with metastatic castration-resistant prostate cancer. Ann Oncol. 2020;31: 619–625. doi:10.1016/j.annonc.2020.01.074

11. Rescigno P, Porta N, Finneran L, Riisnaes R, Figueiredo I, Carreira S, et al. Capivasertib in combination with enzalutamide for metastatic castration resistant prostate cancer after docetaxel and abiraterone: Results from the randomized phase II RE-AKT trial. Eur J Cancer. 2024;205: 114103. doi:10.1016/j.ejca.2024.114103

12. Schmid P, Abraham J, Chan S, Wheatley D, Brunt AM, Nemsadze G, et al. Capivasertib Plus Paclitaxel Versus Placebo Plus Paclitaxel As First-Line Therapy for Metastatic Triple-Negative Breast Cancer: The PAKT Trial. J Clin Oncol. 2020;38: 423–433. doi:10.1200/JCO.19.00368

13. Jones RH, Casbard A, Carucci M, Cox C, Butler R, Alchami F, et al. Fulvestrant plus capivasertib versus placebo after relapse or progression on an aromatase inhibitor in metastatic, oestrogen receptor-positive breast cancer (FAKTION): a multicentre, randomised, controlled, phase 2 trial. Lancet Oncol. 2020;21: 345–357. doi:10.1016/S1470-2045(19)30817-4

14. Howell SJ, Casbard A, Carucci M, Ingarfield K, Butler R, Morgan S, et al. Fulvestrant plus capivasertib versus placebo after relapse or progression on an aromatase inhibitor in metastatic, oestrogen receptor-positive, HER2-negative breast cancer (FAKTION): overall survival, updated progression-free survival, and expanded biomarker analysis from a randomised, phase 2 trial. Lancet Oncol. 2022;23: 851–864. doi:10.1016/S1470-2045(22)00284-4

15. Crabb SJ, Birtle AJ, Martin K, Downs N, Ratcliffe I, Maishman T, et al. ProCAID: a phase I clinical trial to combine the AKT inhibitor AZD5363 with docetaxel and prednisolone chemotherapy for metastatic castration resistant prostate cancer. Invest New Drugs. 2017;35: 599–607. doi:10.1007/s10637-017-0433-4

16. Crabb SJ, Griffiths G, Marwood E, Dunkley D, Downs N, Martin K, et al. Pan-AKT Inhibitor Capivasertib With Docetaxel and Prednisolone in Metastatic Castration-Resistant Prostate Cancer: A Randomized, Placebo-Controlled Phase II Trial (ProCAID). J Clin Oncol. 2021;39: 190–201. doi:10.1200/JCO.20.01576

17. Crabb SJ, Griffiths G, Dunkley D, Downs N, Ellis M, Radford M, et al. Overall Survival Update for Patients with Metastatic Castration-resistant Prostate Cancer Treated with Capivasertib and Docetaxel in the Phase 2 ProCAID Clinical Trial. Eur Urol. 2022;82: 512–515. doi:10.1016/j.eururo.2022.05.019

18. Jhaveri K, Loi S, Hamilton E, Schmid P, Anders CK, Testa L, et al. DESTINY-Breast08: A Phase Ib Study of Trastuzumab Deruxtecan in Combination with Other Anticancer Therapies in Patients with HER2-Low Metastatic Breast Cancer. Clin Cancer Res. 2026;32: 1046–1058. doi:10.1158/1078-0432.CCR-25-0874

19. Smyth LM, Tamura K, Oliveira M, Ciruelos EM, Mayer IA, Sablin M-P, et al. Capivasertib, an AKT Kinase Inhibitor, as Monotherapy or in Combination with Fulvestrant in Patients with AKT1 E17K-Mutant, ER-Positive Metastatic Breast Cancer. Clin Cancer Res. 2020;26: 3947–3957. doi:10.1158/1078-0432.CCR-19-3953

20. Smyth LM, Batist G, Meric-Bernstam F, Kabos P, Spanggaard I, Lluch A, et al. Selective AKT kinase inhibitor capivasertib in combination with fulvestrant in PTEN-mutant ER-positive metastatic breast cancer. NPJ breast cancer. 2021;7: 44. doi:10.1038/s41523-021-00251-7

21. Vaklavas C, Oliveira M, Armstrong AC, Moreno I, Twelves C, Victoria Ruiz I, et al. Camizestrant in combination with capivasertib for women with ER-

positive, HER2-negative advanced breast cancer: results from SERENA-1. ESMO Open. 2026;11: 106049. doi:10.1016/j.esmoop.2025.106049

22. Zhang J, Liu X, Du Y, Mu Y, Meng Y, Sun Y, et al. A Phase I open-label study to assess the pharmacokinetics, safety, and tolerability of capivasertib alone or in combination with paclitaxel in Chinese patients with advanced solid tumors. BMC Cancer. 2025;25: 1562. doi:10.1186/s12885-025-14982-4

23. Schmid P, Ma CX, Park YH, Lord S, Armstrong A, Im S-A, et al. Durvalumab Plus Paclitaxel, with or without Capivasertib or Oleclumab, in Patients with Locally Advanced/Metastatic Triple-Negative Breast Cancer. Clin cancer Res an Off J Am Assoc Cancer Res. 2026. doi:10.1158/1078-0432.CCR-25-4417

24. Yap TA, Kristeleit R, Michalarea V, Pettitt SJ, Lim JSJ, Carreira S, et al. Phase I Trial of the PARP Inhibitor Olaparib and AKT Inhibitor Capivasertib in Patients with BRCA1/2- and Non-BRCA1/2-Mutant Cancers. Cancer Discov. 2020;10: 1528–1543. doi:10.1158/2159-8290.CD-20-0163

25. Westin SN, Labrie M, Litton JK, Blucher A, Fang Y, Vellano CP, et al. Phase Ib Dose Expansion and Translational Analyses of Olaparib in Combination with Capivasertib in Recurrent Endometrial, Triple-Negative Breast, and Ovarian Cancer. Clin Cancer Res. 2021;27: 6354–6365. doi:10.1158/1078-0432.CCR-21-1656

26. Shore N, Mellado B, Shah S, Hauke R, Costin D, Adra N, et al. A Phase I Study of Capivasertib in Combination With Abiraterone Acetate in Patients With Metastatic Castration-Resistant Prostate Cancer. Clin Genitourin Cancer. 2023;21: 278–285. doi:10.1016/j.clgc.2022.11.017

27. Doroshow DB, Shapiro GI, Do K, Keedy VL, Mahdi H, Sohal DPS, et al. Targeted therapy for DNA damage response and homologous recombination repair defects: The Olaparib Combinations trial. Cancer. 2026;132. doi:10.1002/cncr.70332
